# Sustainable Development Goals for Textiles and Fashion

**DOI:** 10.1007/s11356-023-29453-1

**Published:** 2023-09-04

**Authors:** Alka Madhukar Thakker, Danmei Sun

**Affiliations:** School of Textiles and Design, Heriot-Watt University, Edinburgh, UK

**Keywords:** Sustainable Development Goals, Sustainable textiles, Sustainable fashion, UNDP, Climate action, Partnership

## Abstract

In this paper, each of the 17 Sustainable Development Goals (SDGs) is discussed in the context of textiles and fashion. The necessity of collaborative efforts is accentuated to overcome the climate crisis and human health concerns encountered by the textiles and fashion industry. The concerns over poverty faced by cotton farmers, soil depletion, and toxicity to aquatic life due to microfibres and heavy metals are presented. The paper underlines numerous corrective practises such as the utilisation of African Organic Cotton, Better Initiative Cotton, and others that could enable curtailing poverty and hunger. The requirement for a more corporate and socially responsible textiles and fashion business that would propel SDGs is described with inspiring examples of Inditex, Culthread, Saint Basics, Flamingo’s Life, etcetera. More, the greenwashing and ardent necessity of transparency across the fashion value chain is emphasised herein. The importance of reducing inequalities and working in partnership for innovation and justice is highlighted such as apple leather, algal foam, and others. Even more, the production waste and landfill disputes are reviewed. Eventually, the paper concludes with an appeal for mindful and diligent efforts from textiles and fashion consumers, designers, manufacturers, and traders to continually adapt to SDGs even after 2030 as there is no planet B. Also, abiding by the laws of nature as listed herein is found to be the key to reaching SDGs.

## Introduction

The United Nations Sustainable Development Goals is a framework of 17 goals set forth to transform the world. The seventeen goals focusing on global subjects of crisis involve poverty, hunger, good health and well-being, quality education, gender equality, clean water and sanitation, affordable and clean energy, decent work and economic growth, industry, innovation and infrastructure, reduced inequalities, sustainable cities and communities, responsible production and consumption, climate action, life below water, life on land, peace, justice and strong institutions, and partnerships for the goals. The agenda aims to meet the target by 2030, gaining greater peace and prosperity for the people and the planet. The textile and fashion industries are at the heart of SDGs and the European Commission’s Green New Deal as it is worth 2% of the world’s GDB, employ millions of people, and are one of the largest polluters in the world. It generates more than 1.2 billion tons of carbon dioxide emissions, 22 million tons of microfibres, and 21 billion tonnes of waste, 20% of which are extremely toxic wastewater effluent from dye houses (Ellen MacArthur Foundation & Circular fibres initiatives 2017) (The United Nations Sustainable Development Goals 2022) (Textile Exchange n.d.) (SDG Watch Austria 2020). Further scrying reveals that ‘slowing the fashion’ and ‘closing the loop’ are sabotaged by greenwashing. Only 1% of textile is recycled, overproduction by 25% of new garments remain unsold, 35% of the microplastic is leached into the oceans from synthetic textiles, and 100 cubic metres of water is consumed per person for modalities with textiles. Furthermore, recycled polyester is disclosed to be a myth and ultra-fast fashion flow from Shein, H&M, BooHoo, and others will never be sustainable. Amidst obscurity, this paper presents SDGs as a legitimate, viable, and rational framework of action for sustainable textile and fashion industry. Each of the SDGs are demonstrated further in pertinence to the clothing sector: its problems and solutions with SDGs (Cobbing et al. 2023). Conclusively, the overview largely focuses on remedial approaches with SDGs that would propel eco-friendly Future Fashion Factories.

## Methodology

This systematic review abided by a set of protocols as elaborated herein. The aim was to associate each of the SDGs with the textile and fashion industry. Therefore, the published papers reflecting this connection were studied. The literature search largely implemented discovery and google scholar in addition to Scopus and PubMed. The qualitative review paper executed a comprehensive literature search, including case studies, narratives, and authorised reports from the United Nations, European Union, European Food Safety Authority, European Chemical Agency, and others. Additionally, excerpts were analysed, paraphrased, and cited from the journal papers, websites, and literature from reliable sources such as national geographic, textile exchange, and others. Apart from aforementioned keywords, the search terms utilised for the review were sustainable development goals and textile sector and sustainable development goals and fashion industry. Furthermore, each of the SDGs was individually searched in context of textiles, fashion, fabrics, garment, apparel, and other relevant words, for instance, life below water and textile units etcetera. The overview identifies the requirement of answering the research question of how each SDGs append with the textile fashion division? The vital contributions and limitations of the studies reviewed are exhaustively given in this paper in the introduction section and in further text. The paper is meticulously structured to simultaneously provide measures for several issues in the textile and fashion industry based on Sustainable Development Goals (SDGs).

## Literature review

The Sustainable Development Goals (SDGs) are an authorised archetype with an integrated approach that applies to the entire humankind on earth. In 2015, the United Nations Member States set forth the 2030 agenda for sustainable development. The aim is to achieve prosperity and peace for the planet and people by abiding by 17 Sustainable Development Goals across the globe (United Nations n.d.a). Additionally, the Conscious Fashion and Lifestyle Network (CFLN), an online platform, is jointly instigated by the United Nations Office for Partnerships—the Division for Sustainable Development Goals, the Department of Economic and Social Affairs, and the Fashion Impact Fund. The principal objective of CFLN is to propel resources, technology, and innovation in accordance with SDGs for a healthy planet (United Nations n.d.b). This overview pivots on the role of SDGs in catalysing the textile and fashion industry towards Green pathways as it is the second largest polluter on earth after the big Oil industry (Thakker and Sun 2022). This paper comprehensively presents each of the SDGs in relevance to the fabric and clothing sector. Prior to this, authors have elaborated on a few of the SDGs in context of fabric and fashion division such as Vijeyarasa and Liu recommend gender-just fashion sector by infusing SDGs 6, 8, and 12 (Vijeyarasa and Liu 2022). Similarly, SDG 12, responsible production and consumption is emphasised to be most vital for textiles and fashion industries (Gardetti and Muthu 2020). The case studies from the apparel industry, namely, Dutch Awearness, Excess Materials Exchange, Rapanui, Stylelend, and Tejidos Royo recommend reorganising the resources, finances, and business potentials for achieving SDG 12. Additionally, it is crucial to reinforce the circular economy for sustainability in the clothing industry (Gabriel and Luque 2020). Again, Radhakrishnan reinforces SDG 12 and predicts that for a sustainable future of apparels, it is imperative to take a paradigm shift towards customised production, circular fashion, and educate consumers to harness the mindset for ethical and conscious buying behaviour (Radhakrishnan 2020). Likewise, Hempstead accentuates on SDG 12, 14, 10, 5, and 17. The Hempstead review states that the fashion sector utilises 98 million tonnes of non-renewable resources, and our clothing leeches 24 trillion microplastic particles into the world’s oceans causing chaos to marine biodiversity (Hempstead 2022). The mega fashion sector has the responsibility and capability to dynamically reduce social inequalities and gender biases. The world of fashion has the potential to generate 5 trillion dollars of circular economy value by partnership amongst its business corporates, government sectors, and academic institutions (Hempstead 2022). The United Nations Alliance for Sustainable Fashion (UNASF) pivots on clothing, leather, and footwear fashion value chain. It ensures that SDGs are met by coordinated action in the fashion industry from make to finish (United Nations n.d.c). The UNASF states that the clothing industry is responsible for 2–8% of total greenhouse gas emissions, and it eliminates 9% of microplastic into the oceans per year. The UNASF is committed to transforming the fashion industry by implementing outreach and collaborative measures, knowledge sharing, and nurturing the existing synergies (United Nations n.d.a). Hence, numerous vital initiatives are taken by the UN for promulgating sustainability in textiles and fashion. Also, from the above studies, it is deduced that the SDG 12: responsible production and consumption is significantly affecting the ecological index of the fashion industry. However, the textile and fashion industry is vast, and its repercussions on environment and human health necessarily fastens it to all 17 SDGs. It is noted that the textiles and fashion units are rarely associated with the entire framework of SDGs that includes 17 goals at its heart. Consequently, this review paper takes a holistic approach by conjoining each of the SDGs with fashion division.

In the same vein, Textile Exchange (KPMG) opines that the textiles, retail, and apparel sector can contribute to each of the SDGs. Nevertheless, KPMG has distilled 8 SDGs that are vital for these 3 clusters; amongst these, the SDG Goal 13 climate action is of prime concern (Textile Exchange 2018). For instance, accomplishing SDG Goal 13, it would be beneficial to source circular materials for sustainable production and hence reduce the carbon footprints of operations for example GOTS certified brand Tekla, carbon neutral company Hunza G, Arloe, and others. The Kitx is an Australian brand that utilises renewable natural dyes from jackfruit and marigold for colouring its clothing collection named Unearthed Dress (Textile Exchange 2018) (Murray and Jackson 2023). The Textile Exchange recommends achieving SDG 5 gender equality by empowering women and girls by training them for good health, hygiene, sanitation, legal literacy, and others as observed by BITE Studio, Pour Les Femmes, etcetera; SDG 6 is to ascertain safe water and sanitation by zero discharge of hazardous chemicals (ZDHC) in the textiles value chain, recycling waste water, etcetera. The ZDHC and Blue design and Greenpeace dedicatedly work to ensure that the fashion supply chain adapts to best practises observed by brands such as Asket, Kalita, and PRISM Squared. Similarly, SDG 7 emphasises affordable and sustainable energy. Several fashion giants in the market, namely, DL1961 have committed to reducing greenhouse gas emission by implementing renewable sources of energy such as installing solar panels for manufacturing. SDG 8 for sustained economic growth and decent work KPMG suggests following fair trade practises such as giving good work conditions to factory workers including ergonomics, wages, clean water, and food. It would be mandatory to ensure that there is no forced labour across the fashion supply chain. The social certification systems, namely, Fair Labour Association, Fair Wear Foundation, and Cradle to Cradle provide the accreditation for the same (Textile Exchange 2018). Companies and brands such as Veja, Faithfull The Brand, Reformation, and Wolford are certified (Murray and Jackson 2023). SDG 9 focuses on inclusive and sustainable industrialisation, infrastructure, and innovation as endorsed by Lenzing. Lenzing is committed to innovation with sustainably sourced wood, and Petit Pli innovative clothes that grow and shrink with the wearer are eco-friendly. The brand Ninety Percent prioritises new product development from seaweed that is biodegradable (Textile Exchange 2018) (Murray and Jackson 2023). SDG 10 reinforces reduced inequalities amongst countries, especially developing nations that are Africa, India, and others by applying and monitoring occupational health and safety laws and human rights of textile workers. SDG 12 essentially adheres to sustainable production and consumption patterns by recycling, reusing, and repairing the garments and fabrics, for example, brands, namely, We-AR4, Bogdar, Econyl, and Scout (Murray and Jackson 2023). SDG 15, to protect life on land by conserving biodiversity across the entire terrestrial ecosystem for instance, Alohas utilises plant-based leather manufactured from corn and cactus would protect animals (Murray and Jackson 2023). There exists several tools and apps that facilitate the sustainability agenda set forth by SDGs for 2030 as detailed further in this paper (Textile Exchange 2018).

Together, it is observed from the literature review that connecting each of the SDG with the textiles and clothing industry is of paramount importance. As a result, the overview identifies, enumerates, and concludes with adequate remedial measures for achieving on individual SDGs for higher good of Future Fashion Factories as detailed further.

## Results and discussion

As synthesised and distilled from the above, this overview takes an integrated perspective by uniting each of the SDGs with the textile industry. Herein, each of the SDGs is comprehensively discussed emphasising on the ecological challenges encountered by the textiles and fashion sector. Simultaneously, the remedial approaches are listed that would energise the textile industry towards sustainability.

### Goal 1: No poverty

To end poverty in all its forms everywhere by 2030.

#### Issues

**Fig. 1:**
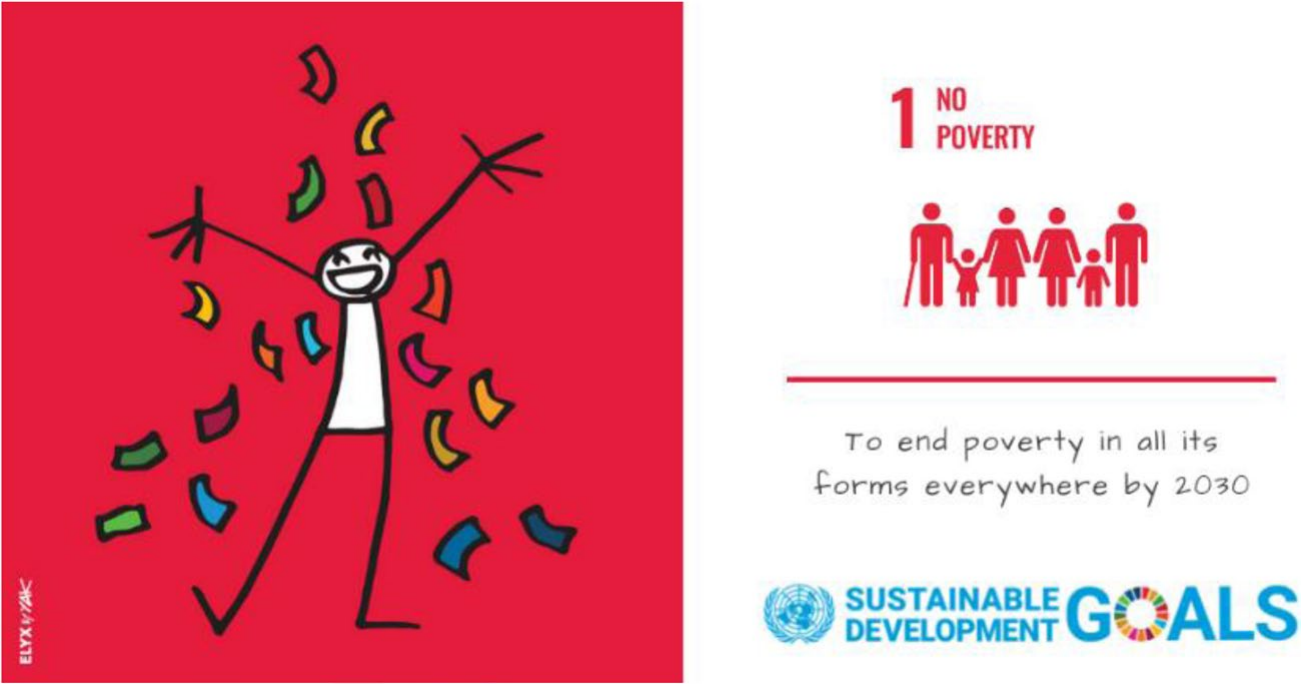
SDG 1, No poverty (The United Nations Sustainable Development Goals 2022)

The garment factory worker’s wages are extremely low leading to poverty. For instance, the high-end garment sold at 300–500 pounds in the UK Luxury wear could have been made at the rate of a maximum of 20–25 pounds gross in India/Bangladesh/Pakistan/China. The larger proportion of garment prices gets reimbursed upon the material, energy bills, and transportation costs. The daily wages of the workers are merely 25–50 pence and therefore insufficient to fulfil their families’ basic needs of food, clothing, and shelter hence, bound to live below the poverty line (Fashion Takes Action 2022) (Luxiders Magazine 2022). It is evident that if the destination points in the supply chain which is a big brand retailer/designer/corporate/consumer practise fair trade, the lost balance would be regained. A brand should transparently indicate how much a 500-pound garment pays back to the daily wage worker who made it (Luxiders Magazine 2022). Even more, in the words of Jennifer Rosenbaun, USA Director of Global Labour Justice: “We must understand gender-based violence as an outcome of the global supply chain structure. H&M and Gap’s fast fashion supply chain model creates unreasonable production targets and underbid contracts, resulting in women working underpaid overtime and working very fast under extreme pressure” (Hodal 2018). By contrast, the Global Living Wage Coalition defines a living wage as: “The remuneration received for a standard workweek by a worker in a particular place sufficient to afford a decent standard of living for the worker and her or his family. Elements of a decent standard of living include food, water, housing, education, health care, transportation, clothing, and other essential needs including provision for unexpected events” (The Global Living Wage Coalition 2023). Minimum wage rules are not in place in manufacturing nations (Vijeyarasa and Liu 2022) (The Global Living Wage Coalition 2023). Likewise, 60% of the cotton is grown by small-scale farmers in developing countries and they are the poorest. They are always in debt and struggle to obtain their food, healthcare, and farming tools. A vicious cycle of poverty could end by persuading sustainability concepts in each mindset involved in the supply chain (Cotton Up 2018). Additionally, culminating in poverty is dumping grounds of discarded clothes such as Atacama Desert in Northern Chile. As a saviour, the startup Ecocitex housed in Santiago manufactures yarns from these discarded clothes. Likewise, EcoFibra innovated padded boards from these textile waste and utilised it for construction purposes. They installed these fabric panels at more than 100 homes in Chile. The United Nations denominated it as a social and environmental emergency (Bartlett 2023). Figure 1 depicts SDG Goal 1—No poverty.

#### Measures

- To raise farmers of cotton, hemp, flax, bamboo trees, and others above the poverty line
- To raise textile factory workers above the poverty line

### Goal 2: Zero hunger

To end hunger, achieve food security and improved nutrition, and promote sustainable agriculture.

#### Issues

**Fig. 2:**
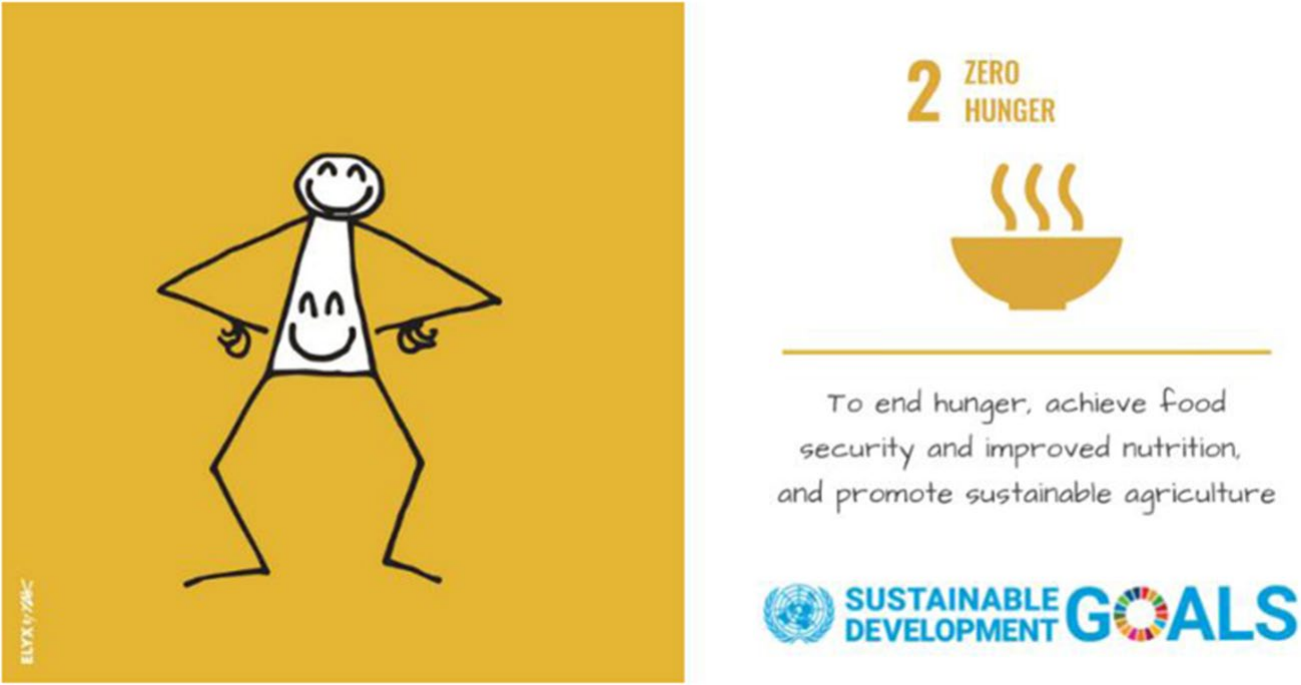
SDG 2, Zero hunger (The United Nations Sustainable Development Goals 2022)

Sequentially, the fashion industry in developing nations must play an active role in enhancing the economic livelihood of the daily wage workers involved in varied departments of production such as embroidery, tailoring, garment finishing, and packing. Fair wages to farmers growing cotton, bamboo, flax, and other fibres and materials for fabric manufacturing and colouring units ought to be paid fair (Fashion Takes Action 2022). Educating small-scale cotton farmers on organic farming including crop rotation and utilising non-GMO (Genetically Modified) seeds for cotton and food would enable them in cultivating food crops and secure sustainable food all around the year. This in turn would improve the quality of land and soil. Figure 2 demonstrates SDG Goal 2—Zero hunger.

#### Measures

- To provide free food to textile factory workers, their children, and families
- To fulfil corporate social responsibilities that eliminate hunger

### Goal 3: Good health and well-being

To ensure healthy lives and promote well-being for all at all ages.

#### Issues

**Fig. 3:**
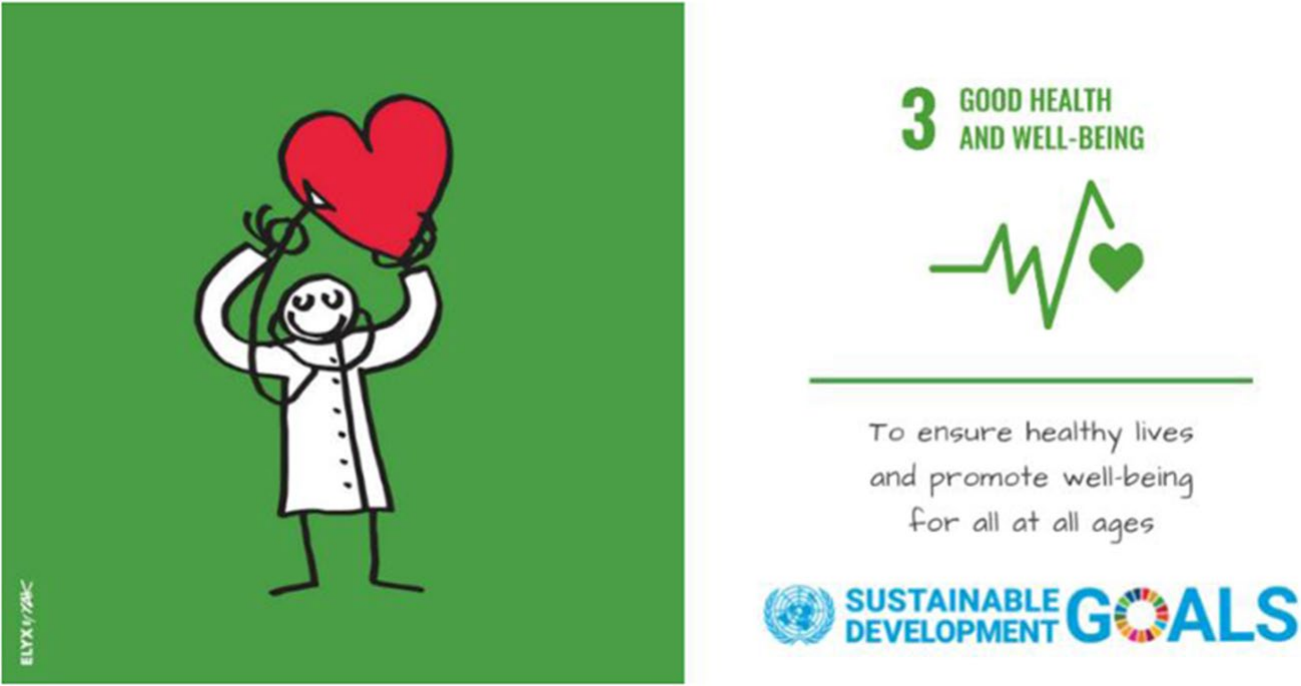
SDG 3, Good health and well-being (The United Nations Sustainable Development Goals 2022)

Curtailing on emissions and effluents implemented in the growing and manufacturing of textiles would improve the quality of health and well-being of the people working and staying in and around that atmosphere (Textile Exchange 2019). Implementing safe material cycles to avoid negative environmental and human health impacts due to the textile sector (Ellen MacArthur Foundation & Circular fibres initiatives 2017). High-risk health and safety conditions and prolonged working hours affecting well-being were observed for a shirt manufacturing company in Bangladesh, a fabric-producing unit in Malaysia, and a spinning site in China (Almanza and Corona 2020). The garment industry scores negatively on SDG 3, indicating the need for massive efforts for improving the quality of life of its workers. Figure 3 illustrates SDG Goal 3—Good health and well-being.

#### Measures

- Phasing out hazardous substances utilised in the growing and processing of textiles
- Textile sectors ought to mandatorily follow health and safety regulations

### Goal 4: Quality education

To ensure inclusive and quality education for all and promote lifelong learning.

#### Issues

**Fig. 4:**
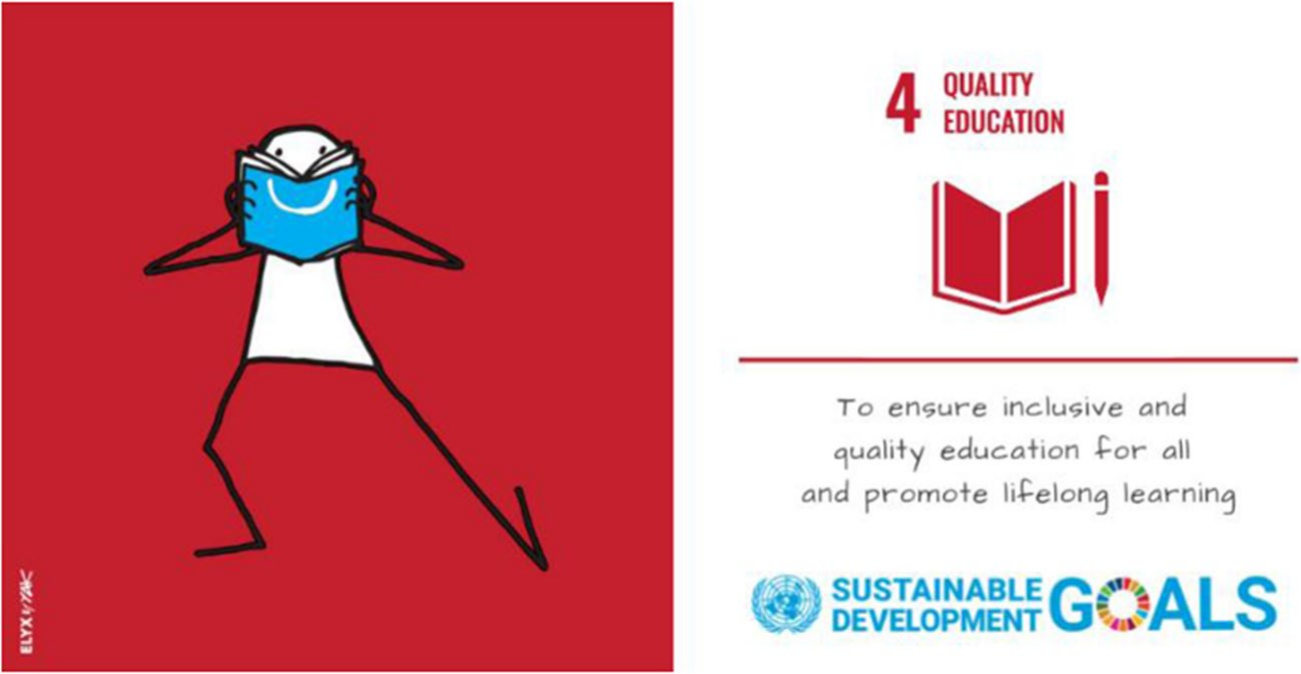
SDG 4, Quality education (The United Nations Sustainable Development Goals 2022)

Synchronously another perspective, for example, Arvind a global leader in apparel and denim manufacturing fulfils its corporate social responsibility by engaging and empowering tribal women. They offer free education and health services to the underprivileged section of society. They have transformed slums into comfortable houses and vertical towers for slum dwellers in Ahmedabad. They work in collaboration with Ahmedabad Municipal Corporation and Swasth India Foundation (Arvind Limited 2017). There are several other like-minded and inspiring manufacturers that align with SDGs. Additionally, using African Cotton is advantageous as its premiums go into the schooling programmes, propelling SDG Goal 4 (Textile Exchange 2019). Figure 4 depicts SDG Goal 4—Quality education.

#### Measures

- To educate fashion farmers on sustainable farming such as crop rotation and herbal fertilisers to prevent soil depletion and increase the harvest
- To educate garment workers about their privileges to wages, work conditions, holidays, perks, and working hours

### Goal 5: Gender equality

To achieve gender equality and empower all women and girls.

#### Issues

**Fig. 5:**
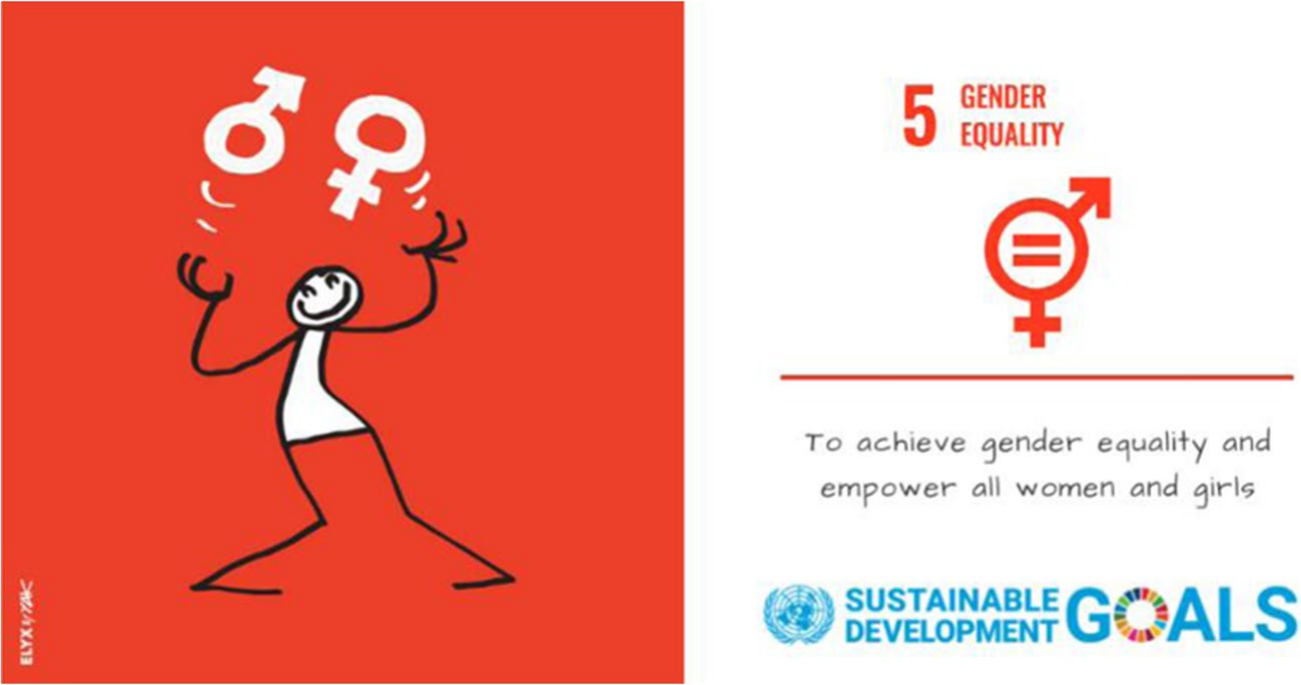
SDG 5, Gender equality (The United Nations Sustainable Development Goals 2022)

It is well known that the textile garment manufacturing units across the developing nations, namely, India, China, Pakistan, Thailand, Mexico, and Vietnam employ women and underage children at lower wages to lower the production cost. All that glitters are not gold for fashion models facing mistreatment (Fashion Takes Action 2022) (Vijeyarasa and Liu 2022) (Malik, et al. 2021). The exploitation could be curtailed by taxation, accountability, maintaining ethics, and giving voice; also, the community system could alleviate gender biases (Vijeyarasa and Liu 2022). There are several social pain points in the fashion supply value chain that require to be resolved. Effective communication and the need for research and development of a standardised methodology to measure and report SDGs would make the organisation more committed to sustainability (Olofsson and Mark-Herbert 2020). Figure 5 shows SDG Goal 5—Gender equality.

#### Measures

- Fairtrade practise
- No child worker
- Work equivalent wage allocation

### Goal 6: Clean water and sanitation

To ensure access to safe water sources and sanitation for all.

#### Issues

**Fig. 6:**
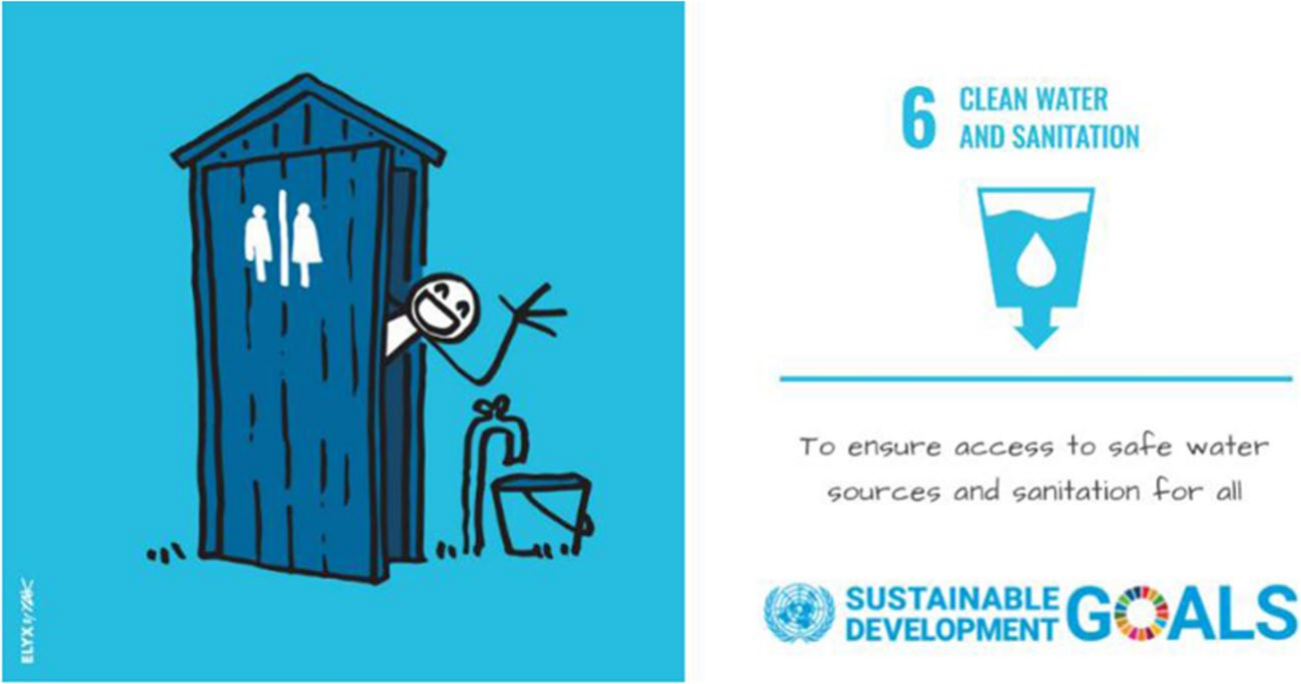
SDG 6, Clean water and sanitation (The United Nations Sustainable Development Goals 2022)

In the same vein, Fairtrade cotton utilises its premiums for providing hygienic toilet facilities for vulnerable and underprivileged populations (Textile Exchange 2019). Similarly, Better Cotton Initiatives have adopted water-efficient cotton cultivation; hence, the freshwater is saved to address water scarcity (Textile Exchange 2019). The bizarre site of tons of fish dead clogging vast river canals would haunt one in sleep (Greenpeace International 2013). The scene repeats for 70% of lakes and rivers in China, namely, the Yangtze River Delta and Pearl River Delta, surrounded by textile colouration units (Greenpeace International 2012). In the same way, the Bandi River, Jaipur (Kakkar 2018), and Noyyal River, Tirupur (Sivasankar 2011) in India are battling pollution from textile colouration sectors. Predominantly, heavy metal toxicity from the colours containing lead, mercury, chromium, copper, ferrous, and cadmium. They are bioaccumulative and damage the human nervous systems and kidneys (Greenpeace International 2012). Clean water is our basic right; however, we are polluting it due to fast fashion that is petroleum-based nature’s wrath is reflected in drought, flood, and acid rain. Hence, implementing alternative recyclable materials such as plant-based metal mordants and additives, natural colourants from hops flowers, and violet herbs (Thakker & Sun, Developing sustainable fabrics with plant-based formulations, 2022) (Thakker, Alka; Sun, Danmei;, Innovative Plant-Based Mordants and Colourants for Application on Cotton Fabric, 2021), adopting Zero Discharge of Hazardous Chemicals (Stichting ZDHC Foundation 2022), and installing an efficient wastewater recycling plant at textile wet processing and colouration units as noted at Banswara Syntex Limited (India) (Banswara Syntex Limited 2016), Yunus Textile Mills Limited (Pakistan) (Yunus 2022), and others. The SDG Goal 6—Clean water and sanitation is given in Fig. 6.

#### Measures

- Fresh drinking water and hygiene facilities for fashion factory workers
- Water recycling plants at textiles wet processing units
- Zero discharge of wastewater by textile colouring units into chief sources of water, namely, oceans, rivers, lakes, and streams

### Goal 7: Clean energy

To ensure access to affordable and sustainable energy for all.

#### Issues

**Fig. 7:**
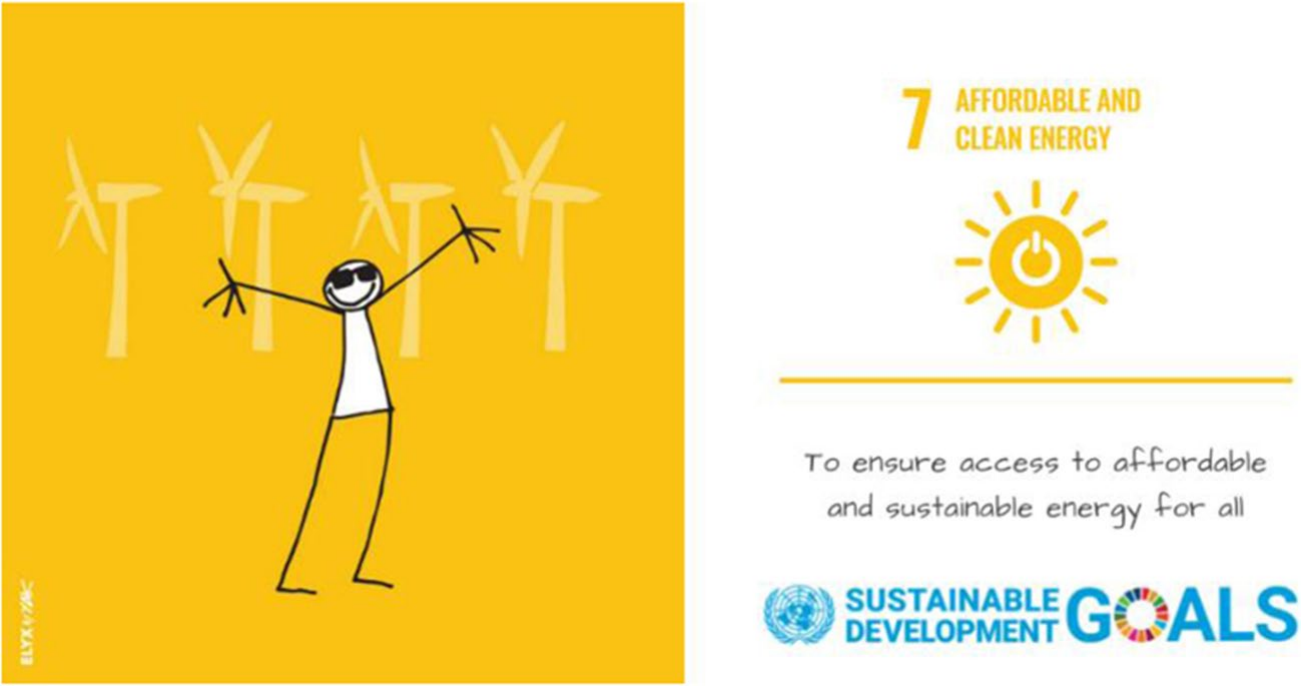
SDG 7, Affordable and clean energy (The United Nations Sustainable Development Goals 2022)

The textile industry is an energy-intensive sector responsible for 10% of total greenhouse gas emissions releasing 1.2 billion tonnes of carbon dioxide per year forecasted to rise by greater than 50% by the year 2030. It is essential to decarbonise and adapt to the green supply chain (Shukla 2022) (The Indian Express Ltd 2021). Synchronously, Debnath reports that fossil-fuel lead boilers at textile flax processing units could be replaced by gas and solar energy-managed boilers, thus reducing the carbon footprints (Debnath 2020). Also, another study revealed that implementing appropriate cleaner practises such as replacing worn-out machines with new ones and utilising efficient technology for instance air jet loom as compared to rapier looms resulted in saving the cotton material cost of USD 9435/month and electric energy cost by USD 39,114/month (Neto et al. 2019). The SDG Goal 7—Clean energy is demonstrated in Fig. 7.

#### Measures

- Textile units to follow the principles of green chemistry for their processing
- Textile mills to instal solar energy plants

### Goal 8: Decent work and economic growth

**Fig. 8:**
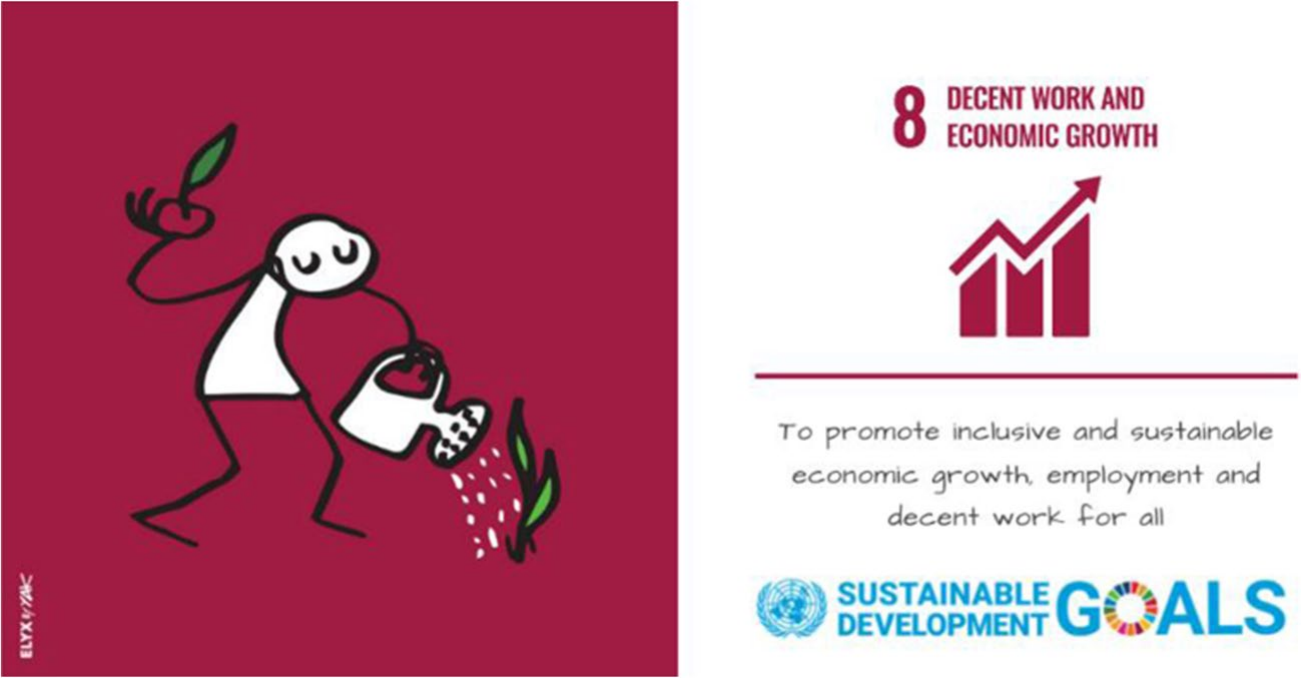
SDG 8, Decent work and economic growth (The United Nations Sustainable Development Goals 2022)

To promote inclusive and sustainable economic growth, employment and decent work for all (Fig. 8).

#### Issues

**Fig. 9:**
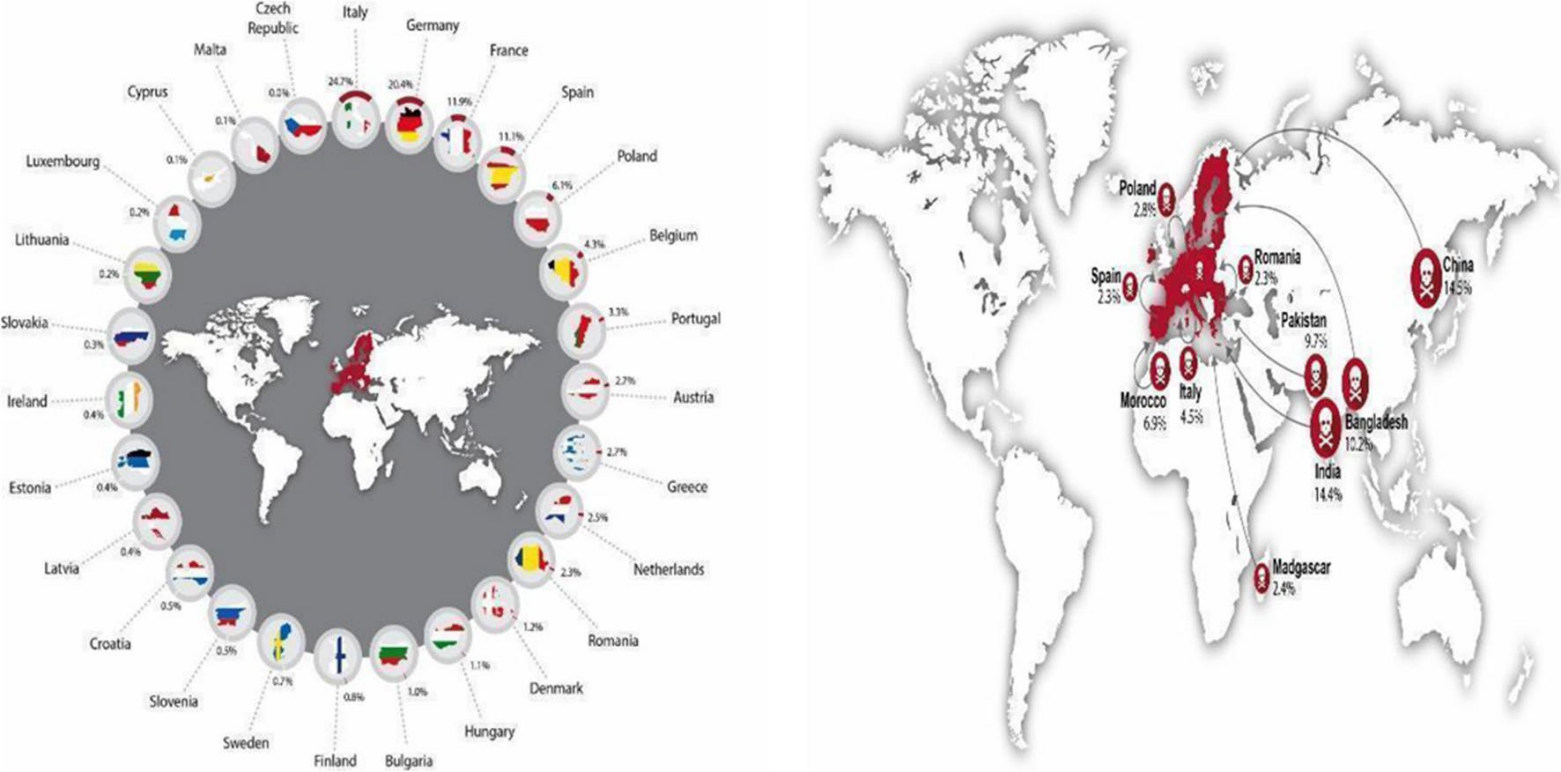
Plotting of fatal accident footprints in the global supply chain to fulfil EU consumption textile demands (Malik, et al. 2021)

An extensive study was conducted on the occupational health and safety of the workers in the textile supply chain to the European Union (EU) covering 15,000 textile units across 189 countries as illustrated in Fig. 9. The research focused on fatal and non-fatal accidents occurring in the aforementioned global supply chain to fulfil the consumption demand of clothing, leather, and other textile products in EU countries as shown in figure (Malik, et al. 2021). It was concluded that Italy, Germany, France, Spain, Poland, Belgium, and Portugal combined are accountable for 80% of the EU consumption-oriented footprint. The multi-regional input–output (MRIO) archive was employed for performing the required calculations. The study aimed to empower in generating decent work and economic growth by eliminating accidents. Overall, 375 fatal and 21,000 non-fatal accidents were reported from the manufacturing units in Madagascar, Romania, India, China, Pakistan Bangladesh, and others as given in figure (Malik, et al. 2021). The mapping of fatal accidents is demonstrated in figures (a and b). A decent work environment addressing the modern slavery terms should be mandatorily applied in extreme-risk garment manufacturing units. Figure 8 shows SDG Goal 8—Decent work and economic growth.

#### Measures

- Health and safety at work including mental, physical, emotional, and financial security
- Respectable wage allocation to fashion workshop workers
- Circular economy

### Goal 9: Industry, innovation, and infrastructure

**Fig. 10:**
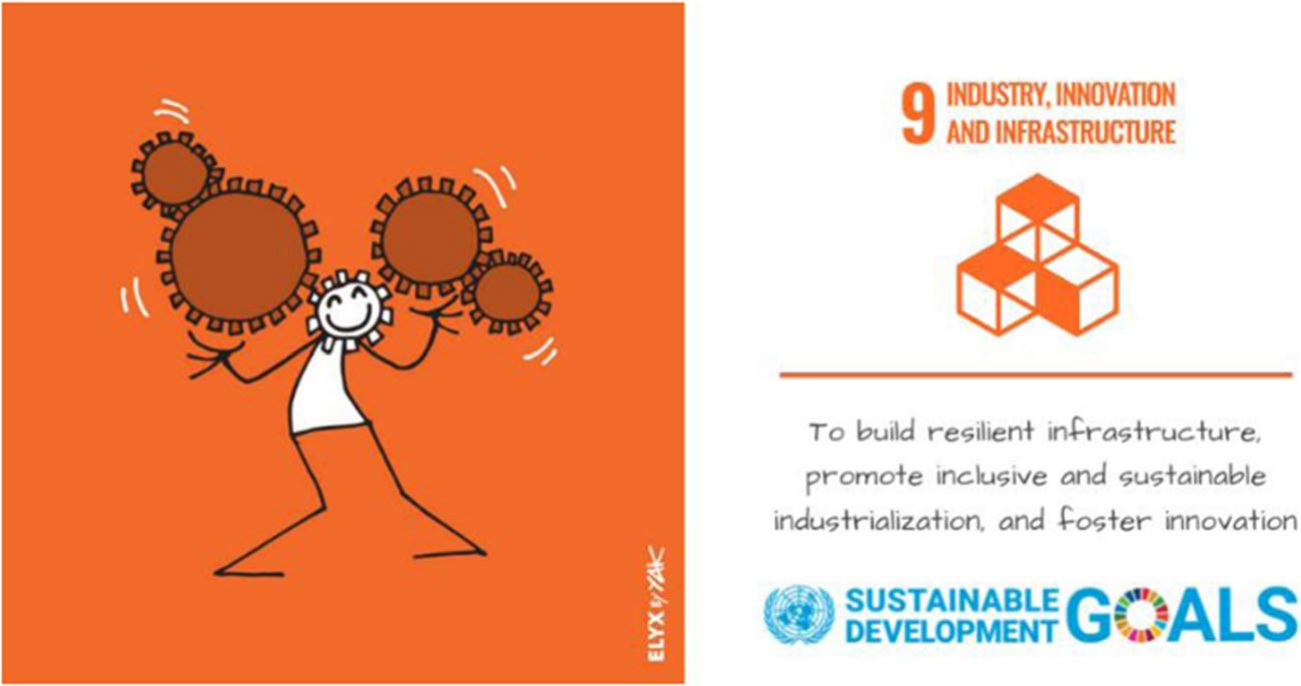
SDG 9, Industry, innovation, and infrastructure (The United Nations Sustainable Development Goals 2022)

To build resilient infrastructure, promote inclusive and sustainable industrialisation and foster innovation (Fig. 10).

#### Issues

**Fig. 11:**
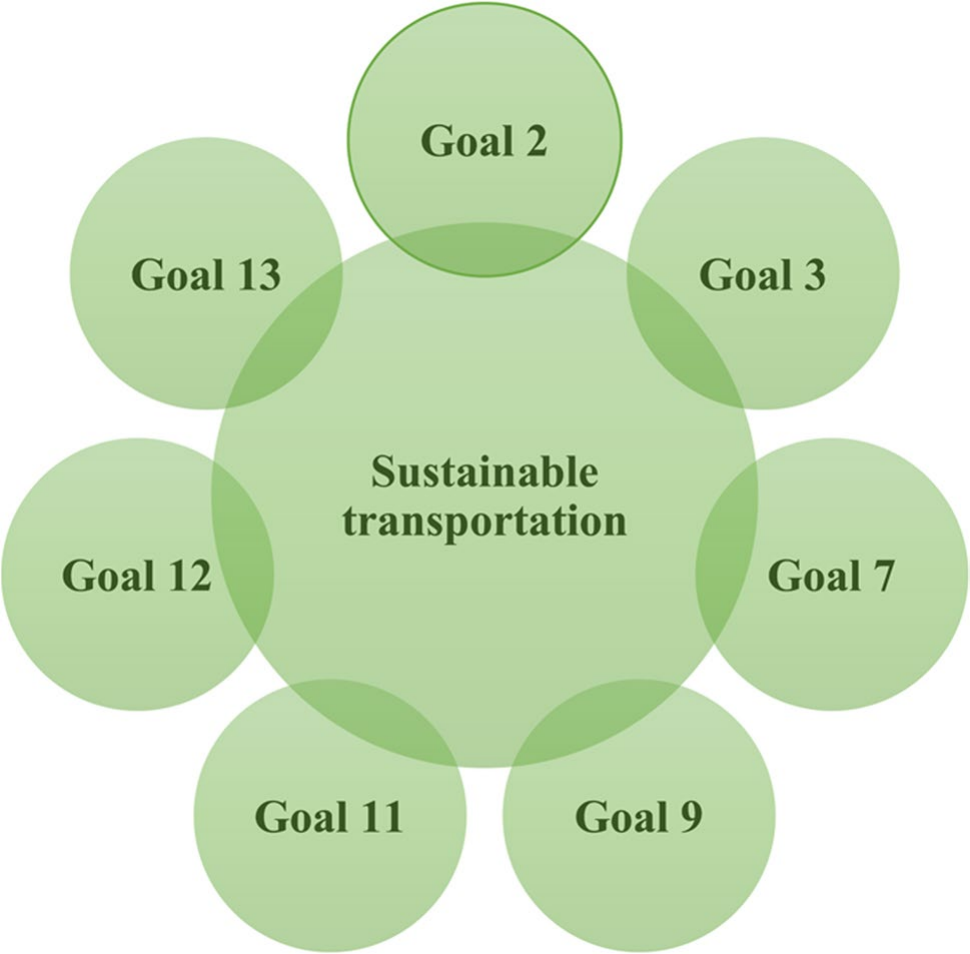
Sustainable transportation chain reaction impacting and benefiting other SDGs (UNDP, High-level Advisory Group on Sustainable Transport 2016)

All the SDGs are interconnected and form a circular nature. In other words, conserving one SDG would produce a chain reaction placing all the SDGs in multiplication mode and thus reaping benefits by manifolds as depicted in Fig. 11, citing an example of sustainable transportation (UNDP, High-level Advisory Group on Sustainable Transport 2016). This phenomenon of chain reaction (Networking) as illustrated in Fig. 11 applies to all SDGs.

In 2014, UN Secretary-General Ban Ki-Moon inaugurated Mobilising Sustainable Transport for Development. The strategies were communicated for all modes of transport, in developing and developed countries (UNDP, High-level Advisory Group on Sustainable Transport 2016). The tactics apply to an entire network of textile and fashion sectors across the world. Sustainable transportation plans and policies would enable the eradication of poverty, encourage financial growth, and propel combating climate change. It was recommended to invest, integrate, and innovate in infrastructure that would enable attaining SDG 9 actively and intelligently (UNDP, High-level Advisory Group on Sustainable Transport 2016). The plight of textile unit workers was evident in the Rana Plaza tragedy in Bangladesh. The calamity reinforces the need for inclusive and sustainable infrastructure. Furthermore, it would be essential to channelise innovations in textiles and fashion to be sustainable from start to finish. Such as, employing waste generated from agricultural and food production can effectively generate ecological fibres and fabrics. For instance, the fabric is made from orange and citrus fruit peels by an innovative patented method even more a cellulosic fabric is further constituted by blending orange and lyocell fibres (Orange Fiber S.r.l. 2022). Synchronously, plant-based leathers are manufactured from cereal crops, pineapple, cactus, corn, apple peels, and flowers by a carbon–neutral process to save animals (Hirsh 2020). An alga is utilised for making foam (Veerah 2022), which is a partially eco-friendly nature-based solution for eco-friendly textiles and fashion. Hence, favour sustainable industrialisation by adapting to ecological methods and methodologies. Additionally, digital innovation models for example knit on-demand models involving manufacturer to the retailer making customised products and roll to bag models wherein 3D-virtual customization of the garment from cut, print, and fit would propel SDGs Goal 9 and 12 (Larsson 2018). Figure 10 illustrates SDG Goal 9—Industry, innovation, and infrastructure.

#### Measures

- Need for safe infrastructure at the textile production site
- Innovation in textiles and fashion to abide by SDGs
- Focus on slow industrialisation for a slow fashion cycle

### Goal 10: Reduced inequalities

To reduce inequalities within and amongst countries.

#### Issues

**Fig. 12:**
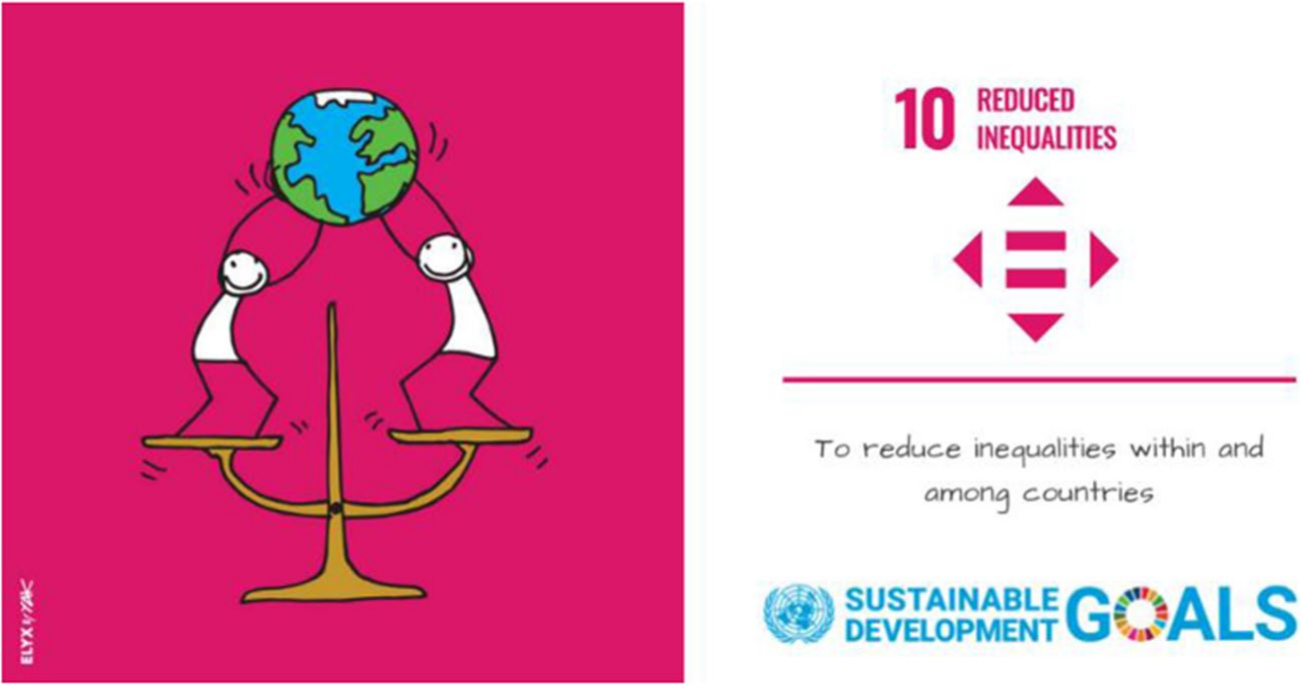
SDG 10, Reduced inequalities (The United Nations Sustainable Development Goals 2022)

The SDG Watch Europe, Wardrobe Change Champaign as depicted in their YouTube video (SDG Watch Europe 2019) demands a radical change in the textile industry. It urges fashion away from the pursuit of cheap clothes and over-economic growth in trade-off to reducing global inequalities (SDG Watch Europe 2019) mounting to human exploitation. Again, the Rana Plaza disaster in Bangladesh, in 2013, triggered a sensitive remark on human rights and democracy. Foreign and Commonwealth Office and Department for International Development, UK, provided 4.8 million pounds for National Action Plan on Fire Safety and Structural Integrity for the garment workers in Bangladesh (Foreign & Commonwealth Office and Department for International Development 2014). Inequalities within and amongst countries raised post-pandemic due to soaring inflation. Sequentially, the cost of materials, namely, cotton, silk, wool, and synthetic fossil-based materials, energy, and transport have sky-rocketed stunting economic growth and adding poverty (Burckel 2022). Figure 12 depicts SDG Goal 10—Reduced inequalities.

#### Measures

- Control inflation in the textile sectors across the nations
- Culminate inequality to BIPOC due to fast fashion landfills

### Goal 11: Sustainable cities and communities

To make cities inclusive, safe, resilient and sustainable.

#### Issues

**Fig. 13:**
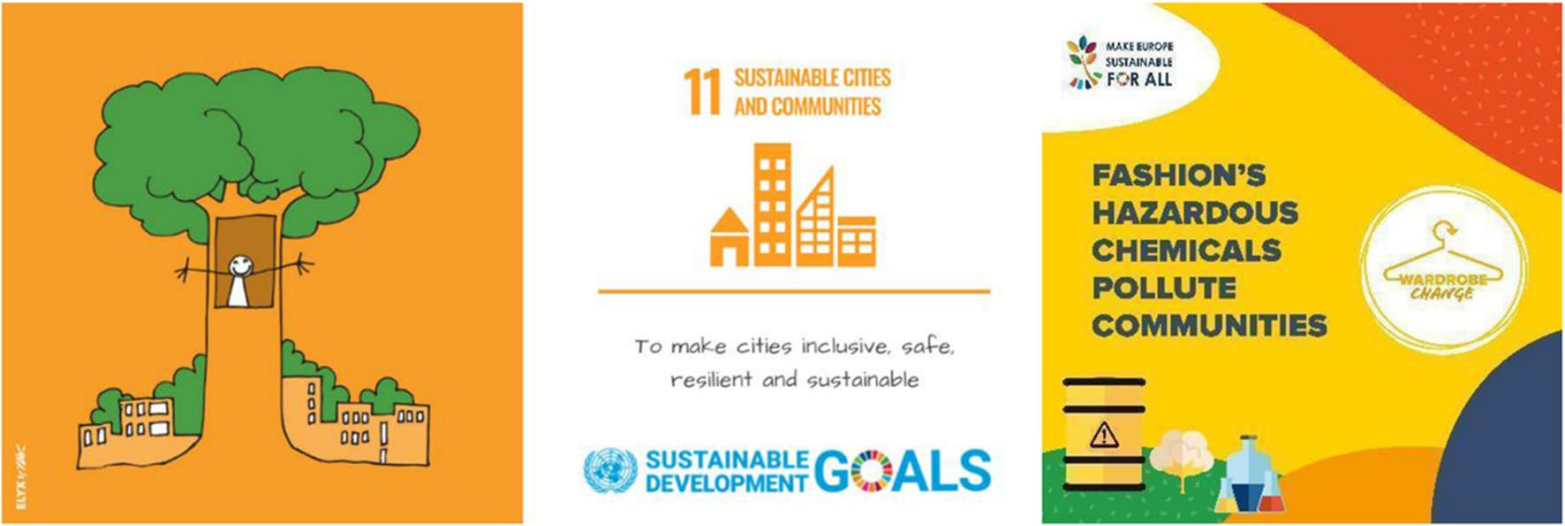
SDG 11, Sustainable cities and communities (The United Nations Sustainable Development Goals 2022) (SDG Watch Europe 2019)

More than 80% of textile waste clothing is dumped in African nations dense in the Black Indigenous People of Colour (BIPOC) communities for example a Kantamanto community in Ghana and several others in Kenya, Malawi, Uganda, Tanzania, Burundi, and Rwanda. Their living conditions are plagued with fires to landfills, un-education, diseases, and poverty (Hajimirsadeghi n.d.) (Earth.Org 2021). On the other hand, there are textile barons such as Inditex (Spain) (Inditex 2022) and Reliance Industries Ltd. (India) (Reliance Industries Limited 2022) who promote community projects reinforcing SDG Goal 11. Another viewpoint is wherein for example the contamination of the Citarum River, Indonesia (Greenpeace International 2013). The textile wet processing unit such as PT Gistex discharges its wastewater into the river Citarum which is utilised by the surrounding farmlands and housing communities. The water is toxic due to high amounts of nonylphenol, nonylphenol ethoxylates, tributyl phosphate, and antimony (Greenpeace International 2013). The SDG Goal 11—Sustainable cities and communities is given in Fig. 13.

#### Measures

- BIPOC communities’ upliftment at textile dumping grounds in Africa
- Corporate social responsibility for sustainable cities and communities

### Goal 12: Responsible production and consumption

**Fig. 14:**
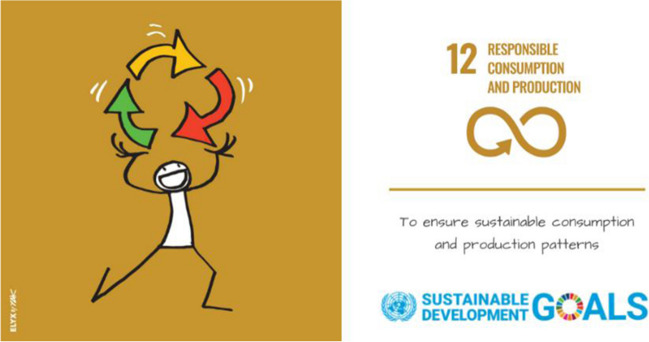
SDGs Goal 12: Responsible production and consumption (The United Nations Sustainable Development Goals 2022)

To ensure sustainable consumption and production patterns (Fig. 14).

#### Issues

**Fig. 15:**
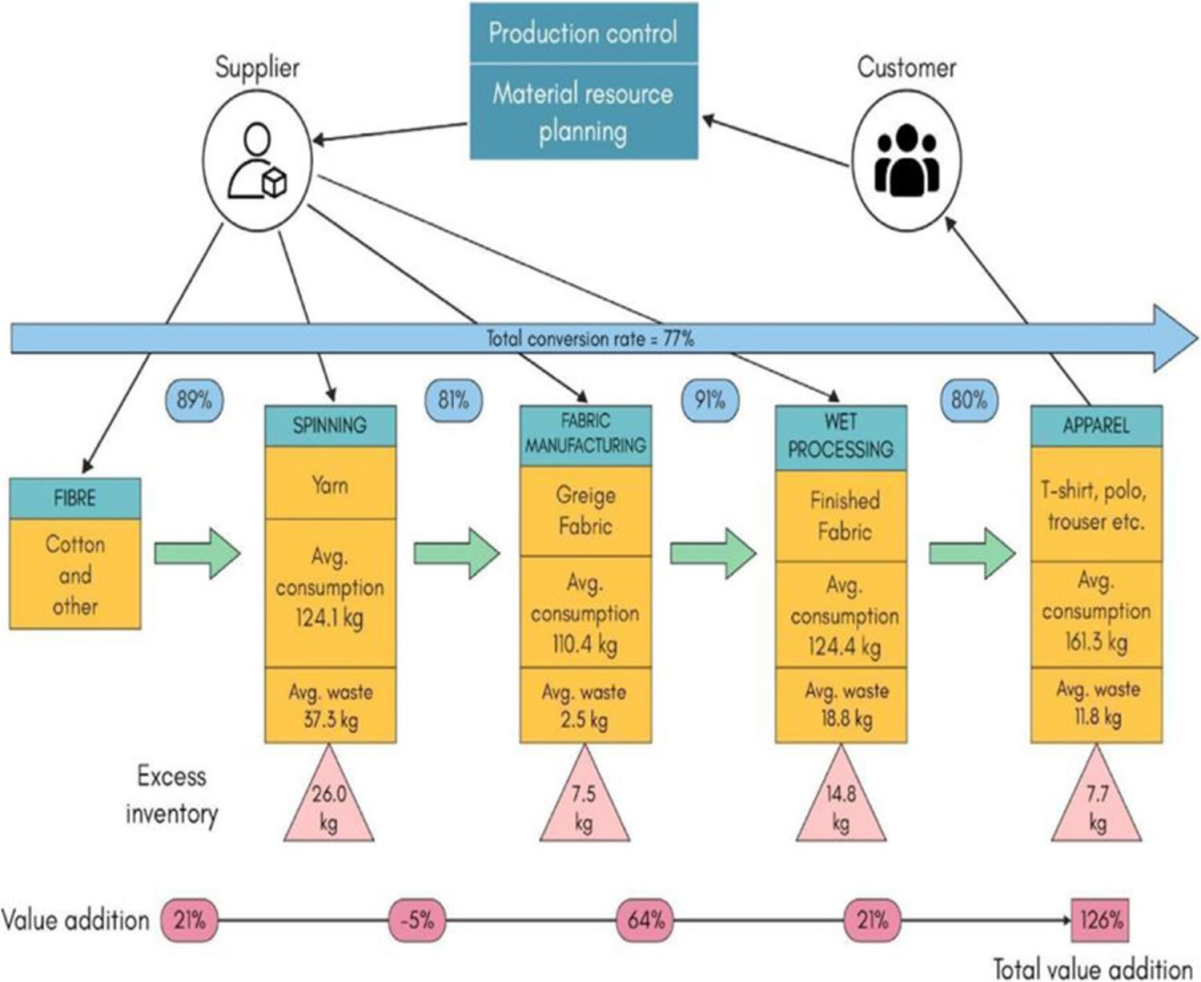
Material stream mapping summarised seventeen Bangladesh textile factories for waste production generating 126% of new value addition

A study was conducted on the production wastage of the 17 textile manufacturing units of Bangladesh; the material stream mapping of which is shown in Fig. 15. It was observed that 126% of more value addition could be generated from the waste produced which amounts to 0.70 USD of gain per piece of production (Akter et al. 2022). A study contributed to SDG’s Goal 12 at the same time propelling a circular economy.

Provin et al.’s comprehensive literature review-based research concluded that the textiles could be upscaled efficiently by reuse, recycling, and refill (Provin et al. 2021). The concept of designing your clothes (T-shirts), for example, Rapanui (Teemill Tech Ltd n.d.) favours sustainability moreover when the garment is worn out; they take it back to recycle it, hence circular. Furthermore, the idea of online shops for second-hand clothing, for example, Vinted (Vinted UAB 2022), Amazon, eBay, Etsy, ThredUP, Vestiaire Collective, Oxfam (Oxfam International n.d.), and others (Threadcurve.com 2021). Also, tailor shops for example as provided by Levi jeans (Levi Strauss and Co 2022) and clothes on rent as offered by Selfridges (Selfridges and Co 2021) could foster sustainability by manifolds mounting to responsible production and consumption goal 12 set forth by SDGs. The overproduction and overconsumption from opulent countries is unshipped in impoverished East African countries, namely, Uganda, Tanzania, Kenya, and Rwanda. These clothing bundles known as ‘Mitumba’ are camouflaged as donations and gifts to those deprived regions in-real poisoning environment and human health. Herein, modern colonialism is apparent that erodes the basic rights of poverty-stricken communities to clean and safe living conditions (Cobbing et al. 2022), hence necessitating the SDG Goal 12 for responsible production and consumption. Figure 14 demonstrates SDG Goal 12—Responsible production and consumption.

#### Measures

- Designers and manufacturers vow for utilising biodegradable materials only
- Consumer awareness to buy less and enjoy more to prevent overflowing landfills
- Manufacturers ought to avoid over-sourcing, over-processing, and over-production

### Goal 13: Climate action

To take urgent action to tackle climate change and its impacts.

#### Issues

**Fig. 16:**
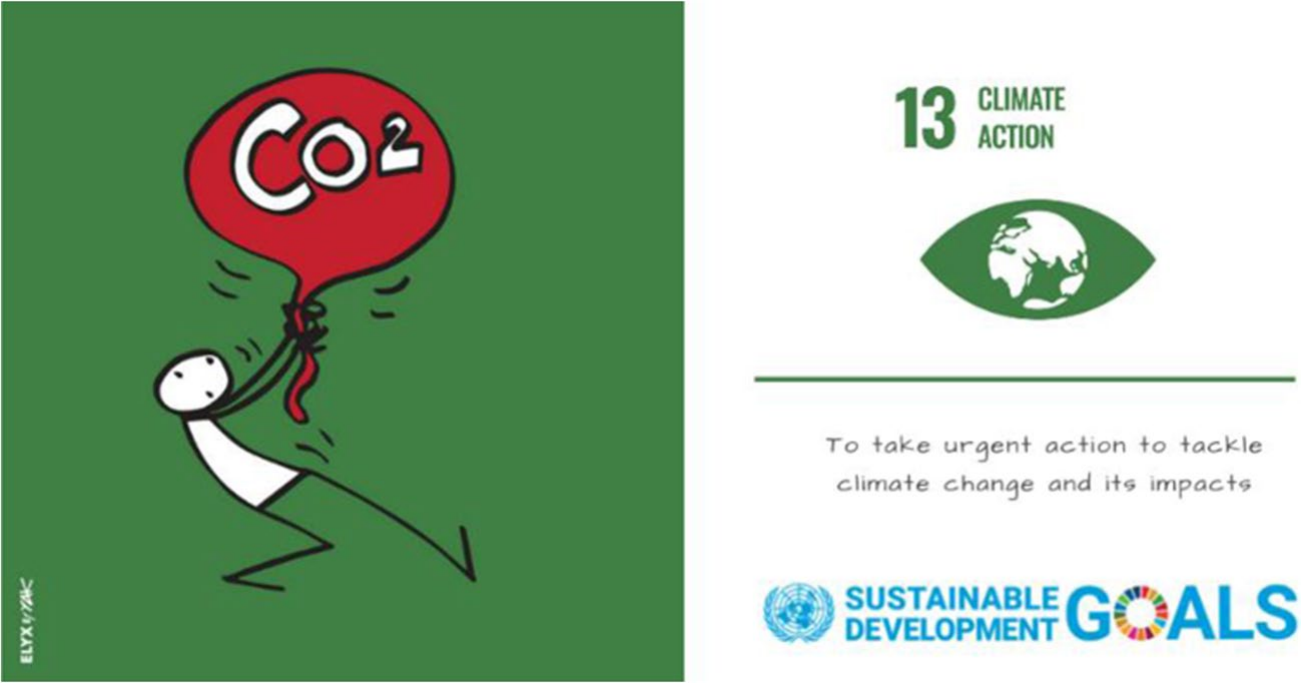
SDG 13, Climate action (The United Nations Sustainable Development Goals 2022)

Figure 16 shows SDG Goal 13—Climate action. Volatile organic compounds (VOCs) from the textile print industry are hazards [28]. According to a United Nations Environment Protection Agency study, the textile printing industry eliminates 99% of its total Toxic Release Inventory into the air, whilst the outstanding one percentage of discharges is let into water and land at a 50–50 ratio, respectively. Typical VOC emissions per textile print line are 130 Mg (megagram)/year (143.3 US ton) for roller prints and 29 Mg/year (31.967 US ton) for flat and rotary screen prints (1 Mg = 1000 kg). Since 1995, greater than 41 million pounds of toxic complexes were released into the environment by the printing industry in the United States alone; the statistics have grown manifolds by now (OEcotextiles n.d). The ten significant chemicals identified as the most polluting VOCs from the textile print industry are given in Table 1; they are all petroleum derivatives. Their hazards to the environment and humans are succinctly informed in Table 1.

**Table 1:** Review of dangers of volatile organic compounds emitted from the textile print industry (Christie et al. 2000) (OEcotextiles n.d.) (European Chemicals Agency 2017) (European Food Safety Authority 2021) (Marrion 1994)

Chemicals	Human hazards	Environment risks
Toluene	Causes nausea, headaches, unconsciousness, and even death	Affects unborn animals, causes membrane damage to plant leaves, and is toxic to aquatic life
Ethylene glycol mono-n-butyl ether, all glycol ethers	The central nervous system, depression, headaches, lethargy, weakness, slurred speech, staggering, tremors, blurred vision, and personality changes, and cause cancer in laboratory animals	Toxic to aquatic life, greenhouse gas effect, and global warming
Methyl ethyl ketone	Adverse effects on the nervous system include headaches, faintness, vomiting, and coldness in the fingers and toes to unconsciousness, liver, and kidney problems	Forms photochemical smog, greenhouse gas effect, and global warming
Xylene	Causes headaches, dizziness, vertigo, and drowsiness. Large amounts of xylene could result in breathing problems, damage to the liver and kidneys, loss of consciousness, heart failure, and death	Ozone depletion, smog formation, greenhouse effect, and global warming
Methanol	Drowsiness, a reduced level of awareness (CNS depression), confusion, pain, dizziness, and the inability to coordinate muscle movement (ataxia)	Affect animals, birds, and fish, leading to their death, and the small growth rate in plants affects the fertility of biota
1,1,1, Trichloroethane	Narcotic effect	Ozone depletion thereby causes skin cancer and eye cataracts
Dichloromethane	Numbness, coldness, pain, and burns to skin and eyes	Deprives the body of oxygen, animals, and humans
Ethylene glycol	Affects the central nervous system (CNS), then the heart, and finally the kidneys	Toxic to aquatic life, microorganism, and microalgae
Ketone	Narcotic or anaesthetic effects	Ozone depletion and photochemical smog
Titanium dioxide	Carcinogenic to humans	Toxic sludge harms marine life

Solvents are utilised in high amounts in the textile print industry and pose dire consequences, including abnormal chromosomal changes. The Registration, Evaluation, Authorization of Chemicals (REACH) and Environment Protection Agency (EPA) have banned several of them; however, they are still manufactured and utilised due to selfish vested interests (OEcotextiles n.d.). All at once, urea (CH_4_N_2_O) is also massively utilised in the textile print industry as a humectant to enhance the wettability of cotton fabric. Nevertheless, synthetic urea poses environmental concerns due to its consumption in large amounts (Christie et al. 2000) (Shore 1990). Subsequently, a hydrotropic product, polyethylene glycol (PEG), was recommended to substitute urea by 70%, preserving the performance of printing paste for cotton fabrics. On the contrary, PEG compounds and petroleum derivatives were observed to be carcinogenic, exhibiting chromosome aberrations and systemic toxicity (David Suzuki Foundation, One nature 2022) (Nontoxic Certified 2019) (Biondi et al. 2002). It is essential to refute the dilemmas at once and support SDG 13 concerning climate change due to fast fashion.

#### Measures

- Ozone depletion, smog formation, greenhouse effect, and global warming due to textile industries and fast fashion
- Reduce carbon footprints from textile emissions and effluents and address the climate emergency

### Goal 14: Life below water

To conserve and sustainably use the world’s oceans, seas and marine resources.

#### Issues

**Fig. 17:**
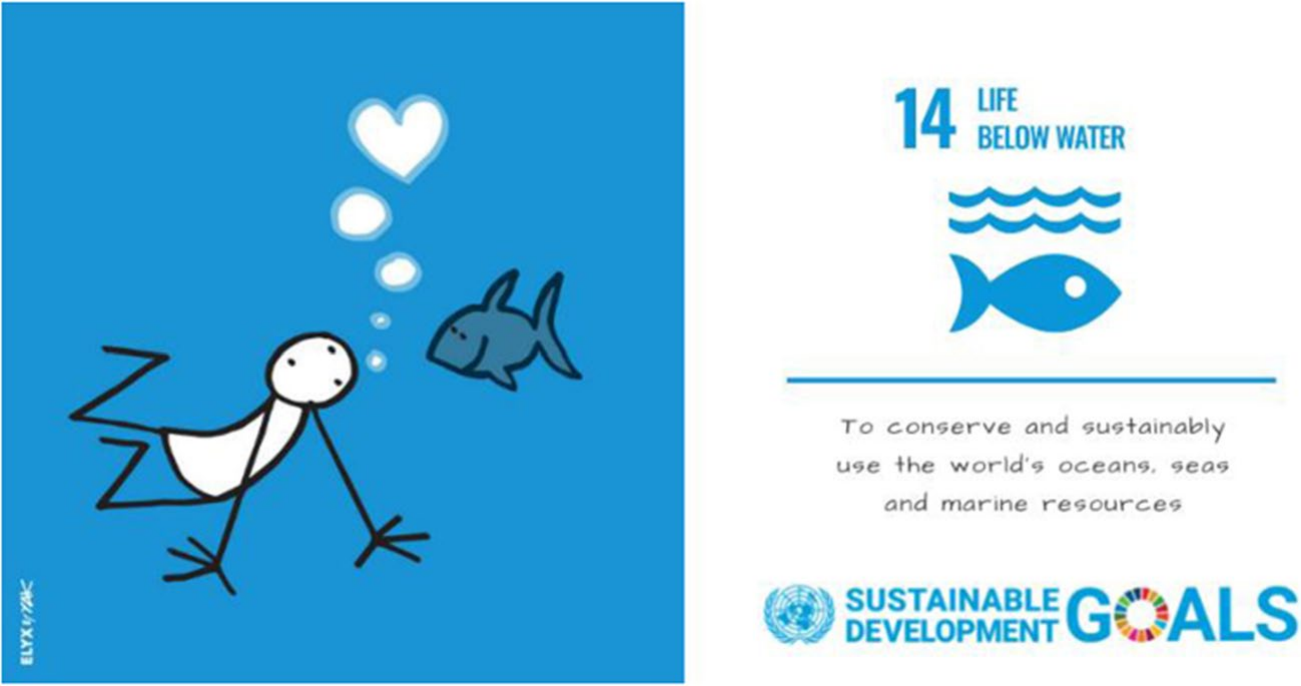
SDG 15, Life below water (The United Nations Sustainable Development Goals 2022)

Microfibres from nylon and polyester fabrics are found in oceans, shellfish, tap water, and others. Microfibres enclose phthalates and plasticisers pollute the air and enter the lung system leading to diseases such as asthma and cancer. It was reported that a finished textile garment releases 175 and 560 microfibres/gramme (Belzagui et al. 2019). The International Union for Conservation of Nature calculated that 35% of microplastics are generated through the laundering of polyester textiles (Maiti 2022). Plastic Soup Foundation testified that 92% of the total plastic originate from textiles, namely, carpets, upholstery, and clothing (Sánchez 2020). Figure 17 demonstrates SDG Goal 15—Life below water.

#### Measures

- A wastewater treatment plant is a must at each textile manufacturing and processing unit
- Protect aquatic biota from synthetic toxic chemicals and microfibres

### Goal 15: Life on land

To sustainably manage forests, combat desertification, halt and reverse land degradation, and halt biodiversity loss.

#### Issues

**Fig. 18:**
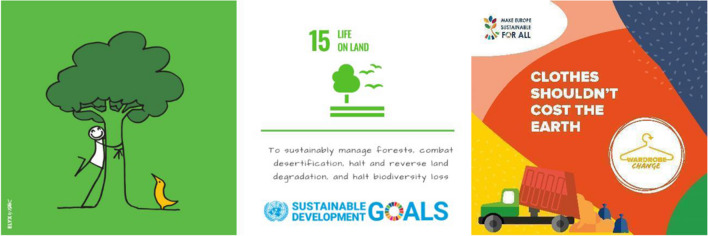
SDG 15, Life on land (The United Nations Sustainable Development Goals 2022) (SDG Watch Europe 2019)

Due to fast fashion each year, more than 85% of textiles go to landfills in Beirut, Ghana, or others (Earth.Org 2021) (McFall-Johnsen 2019). Kantamanto Market in Ghana receives 15 million clothes every week and recirculates 100 million garments every four months. The Kantamanto community is always in debt and is forced to spend on buying landfills at the expense of their healthcare and education system (Earth.Org 2021). Plant-based materials are utilised especially for textile colouration, for example, turmeric, madder root, indigo, nettle leaves, dandelion leaves, violet herb, hops flowers, oak bark, quebracho red, the fame of forest flowers, and several others. Similarly, fibres to fabric production from plants such as bamboo, cotton, linen, hemp, and stinging nettle is promulgated worldwide (Thakker and Sun 2022) (Debnath 2015). Components from the plants are biodegradable and renewable; therefore, the consumer of it must take the responsibility of planting them back for continued harvest and to prevent the plant species from extinction. Crop rotation and employing herbal pesticides would greatly benefit in preventing soil depletion and land erosion (Thakker and Sun 2021). Figure 18 shows SDG Goal 15—Life on land.

#### Measures

- Landfills due to textile wastes ought to be addressed at once
- Plant trees twice the amount of consumption by the textile sector

### Goal 16: Peace, justice, and strong institutions

To promote peaceful and inclusive societies, provide access to justice for all, and build effective, accountable and inclusive institutions at all levels.

#### Issues

**Fig. 19:**
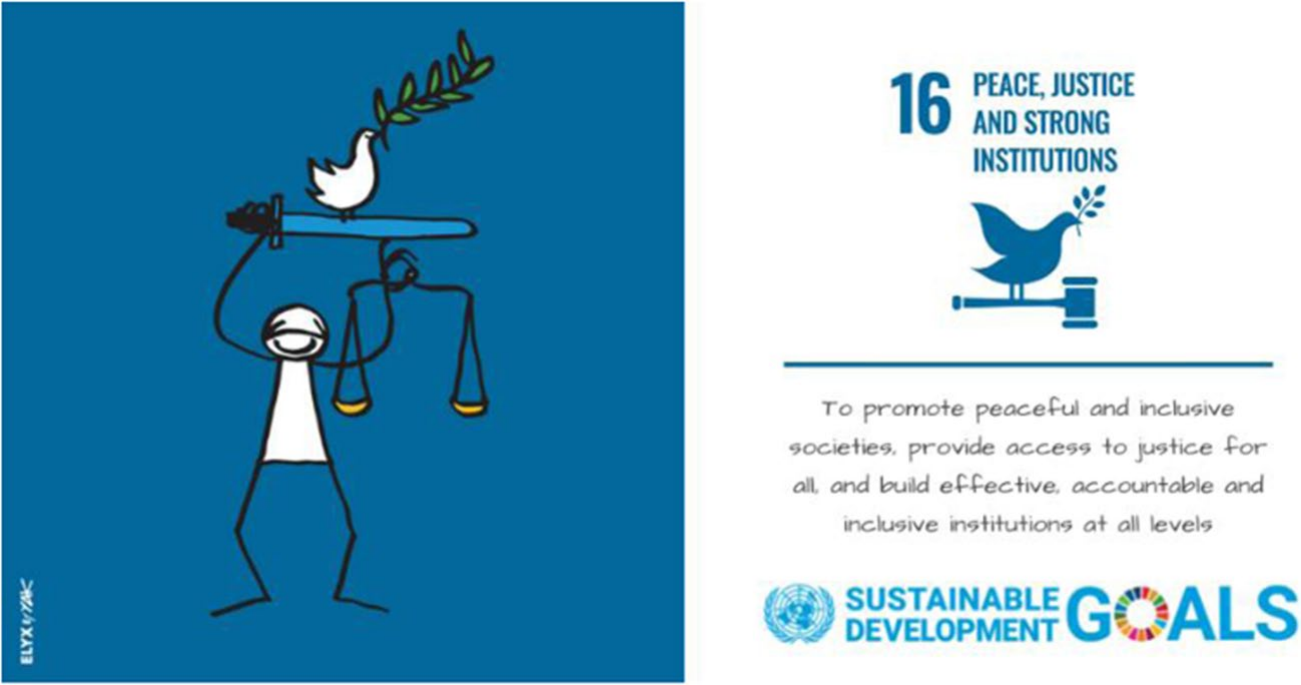
SDG 16, Peace, justice, and strong institutions (The United Nations Sustainable Development Goals 2022)

Goncalves and Silva in their intensive literature-based study on transparency in the apparel industry whilst scoring for sustainability in environmental footprints and social impacts concluded that it is vital to reduce greenwashing that is false and obscure claims of being sustainable and eco-friendly (Robinson 2021) (Gonçalves and Silva 2021). They observed loopholes in communication from the manufacturer (make) to the consumer (finish) leading to a lack of accountability and justice. For instance, amongst the 10 retailers in the study, only 30% communicated about the Modern Slavery Initiative likewise the living wage and wellbeing initiatives (Gonçalves and Silva 2021). Fashion brands, namely, H&M, Primark, Shein, Zara, Boohoo, Asos, Dior, Fendi, and Celine inflict injustice on animals and make fabricated claims utilising fluffy language and green terminology such as eco-friendly thereby hampering SDG Goal 16 (Wolfe 2022) (Igini 2022). For a peaceful and inclusive society, it would be essential to do justice and be honest at all levels of the fashion supply chain. Parallelly, there are solutions providers such as Project CECE (Project CECE 2016), Good on You (Good On You 2022), and Ethical Made Easy (Ethical Made Easy 2020) to enable consumers to combat and refute the brands that pretend to be sustainable and hence positively reinforce the agenda outlined in SDG Goal 16. The examples of animal cruelty-free, eco-friendly, and sustainable brands are Ralph Lauren, Prada, Burberry, Honest Basics, Reer 3, Good Guys, The Nude Label, and several others as enlisted in the directory and brand ratings displayed by good on you and ethical made easy (Ethical Made Easy 2020) (Wolfe 2022) (Robertson 2021). The SDG Goal 16—Peace, justice, and strong institutions is shown in Fig. 19.

#### Measures

- Textiles factories and fashion brands to ensure commitment and investment in circularity programmes and transparent business
- Mandatorily adapt to fair trade business models to eliminate child abuse and corruption in the textile sector

### Goal 17: Partnerships for the goals

To revitalise the global partnership for sustainable development.

#### Issues

**Fig. 20:**
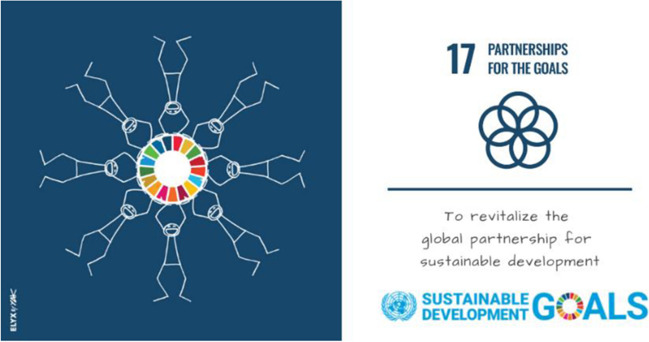
SDG 17, Partnerships for the Goals (The United Nations Sustainable Development Goals 2022)

Moreover, trade and global value chains were provided with 1.8 million pounds to foster partnerships in-between buyers, factory owners, civil society, and others to develop garment factory workers’ conditions in Bangladesh (Foreign & Commonwealth Office and Department for International Development 2014). Diverse mindsets are working towards propelling sustainability in textiles such as C. Collet, a bio designer who engages in growing mycelium with different patterns that could replace petroleum-based binding agents and tie-dye materials and processing. In the same vein, bacterial cellulose is cultivated and widely researched for application in the textile, medicine, food, and paper industries (Wood 2019). The processing is performed at ambient temperature and materials are biodegradable. A field of Bio design, material science, textiles, and fashion are collaboratively working fulfilling SDG Goals 9, 12, 13, and 17. For people, the planet, and profit, the closed-loop fashion supply chain management is recommended as an efficient approach that supports the circularity of materials and the economy. However, to achieve SDGs goals that not only focus on responsible production and consumption but also prioritise culminating poverty and inequalities, it would be mandatory for the stakeholders, namely, the governance, manufacturers, and designers to work in partnerships with the retailer, consumer, recycler, NGOs, and investors (Cai & Choi 2020). A vicious network circular in nature is perceived that would assist in achieving Sustainable Development Goals. The fashion designer Stella McCartney proposes a life cycle thinking approach starting with design and raw materials, production, distribution, use, and end-of-life of the garment to reduce its environmental and social impacts. Sustainable business models and industry tools such as The EcoMetrics, The Kering Environmental Profit and Loss tool, and The Higg Index enable us to investigate and calculate the whole life cycle (Alison 2020). Similarly, it is suggested to rethink business models such as collaborative consumption models, co-operatives, not-for-profit social enterprises, and B-corps (Sharpe et al. 2022). Figure 20 demonstrates SDG Goal 17—Partnerships for the goals.

#### Measures

- Textile manufacturers, designers, consumers, traders, and investors are required to work collaboratively
- Global partnership to address, commit, and report on SDGs followed by textile sectors

Together, the overview of 17 SDGs is assiduously organised herein featuring issues on raise in textiles division. In summary, the highlighted measures would propel the fashion industry in achieving each of the SDGs. Furthermore, the review paper extends diverse alternative routes to synchronise with SDGs as detailed further.

## Application of SDGs for textiles and fashion at home

Handy tips for sustainable care of textiles and fashion at home are listed as follows.

1. Reuse unwanted clothes by selling them on Vinted and others (Vinted UAB 2022)
2. Consumer awareness on curtailing impulsive buying promulgated by fashion magazines and social media networks and apps such as TikTok and others need to be regulated (The Economist Newspaper Limited 2022). Instead, fashion customers could download and link with eco-friendly fashion awareness and renting apps, namely, Greenpeace International, Ellen Macarthur Foundation, Depop, Rent the Runway, Good On You, and several others (The Economist Newspaper Limited 2022)
3. Recycle old clothes in exchange for new clothes (Teemill Tech Ltd n.d.)
4. Refill laundry detergent plastic bottles with natural soap nuts or eco-friendly liquids. Eco-friendly laundry soap such as a reetha nut aka soap nuts and shikakai must be utilised to combat synthetic detergent perils to the ecosystem (Thakker, Sustainable processing of cotton fabrics with plant-based biomaterials Sapindus mukorossi and Acacia concinna for health-care applications 2021). Several other eco-friendly laundry liquids are available, namely, Ecover laundry liquid zero and Koala eco natural laundry liquid (Hello Natural Living 2021)
5. Microfibres at home could be controlled by consistent ventilation for air circulation and vacuum cleaning (Sánchez 2020)
6. Wear clothes for longer duration and cold wash at short cycles as it would prodigiously save on vital resources such as water and energy (Patwary 2020)
7. Buy less and enjoy your clothes more to prevent landfills. It is suggested that each person should reduce their clothing purchase by 75% and value clothes (Sharpe et al. 2022)
8. Consumers can support slow fashion brands, namely, Olivia Rose, The Label, Theory, Amlul, Pringle of Scotland, Daughter, Mary, and likewise. These brands highlight seasonless collections, customised and small quantity production, handmade products, and genderless styles. It reinforces slow fashion (Murray and Jackson 2023)
9. Buy clothes from shops and brands that carry certification of sustainability such as Ecosia (Ecosia 2017) and UNDP shop (The United Nations Development Programme (UNDP) 2022)
10. Beware of greenwashing textile corporations and fashion brands
11. Avoid cheap petroleum-based clothes to refute fast fashion
12. Check for certifications such as Fair Trade Certified, Global Organic Textile Standard, Good weave, Oeko-Tex, Cradle-to-Cradle, and CO2 Logic

Hence, awareness at individual level is imperative to impel SDGs for eco-textiles. Cumulatively leading to much-needed mass revolution for green fashion.

## Conclusions

The United Nations Sustainable Development Goals is a holistic framework outlined in 2014 and is determinedly working towards meeting the set agenda by 2030. There is no question that SDGs have mobilised the world and realise that we are one. There are ample resources by which we can act now for the higher good such as the United Nations Act Now (United Nations n.d.b), Samsung Global Goals (Samsung Global Goals n.d.), and Global Mindpool (The United Nations Development Programme n.d.). The review paper methodically identifies and conjoins each of the Sustainable Development Goals with the textile industry for higher good of the future fashion factories. The crucial issues and their remedial measures are apprehended parallelly for ease to the reader of this review paper. Application of SDGs for textiles and fashion at home as given above could be readily adapted. Moreover, the authors strongly recommend to uphold and follow the laws of nature and vouch for the takeaway message as mentioned further for addressing the climate crisis due to ultra-fast fashion. This paper acknowledges several textile companies and fashion brands that are eco-friendly. The importance of SDG 12 of responsible production and consumption for the apparel industry is highlighted.

Additionally, the SDG framework must continue to be adapted, followed, and monitored post-2030 intended for the eco-friendly, healthy, and prospering lifestyle of future generations to come. All the sectors to mention primarily food, clothing, and shelter are interconnected and circular. Equally, the SDGs apply to all sectors and therefore collaborative efforts are understandably needed. The journalist and professor of climate justice Naomi Klein in her book ‘No Logo’ makes a standpoint that developing nations are considered viable for garment production because of cheap labour and lenient rules and regulations. She aims at brand bullies including fashion brands such as Nike and others highlighting ruthless corporatism. A clear perspective is presented on tackling the concerns from the grassroots level for climate justice (Klein 2019).

Overall, it is imperious to connect with the laws of nature such as listed below are the twelve laws of nature (Oldale 2019).

1. The law of oneness (source is one)
2. The law of vibration (energy levels)
3. The law of action (karma)
4. The law of correspondence (what is inside gets reflected outside)
5. The law of cause and effect (action-reaction to get balance)
6. The law of attraction (connection)
7. The law of compensation (you reap what you sow to get equilibrium)
8. The law of perpetual transmutation (change is constant)
9. The law of relativity (good is valued due to the bad)
10. The law of polarity (yin and yang)
11. The law of rhythm (patterns and cyclic nature)
12. The law of gender (Prakriti and Purusha)

**Fig. 21:**
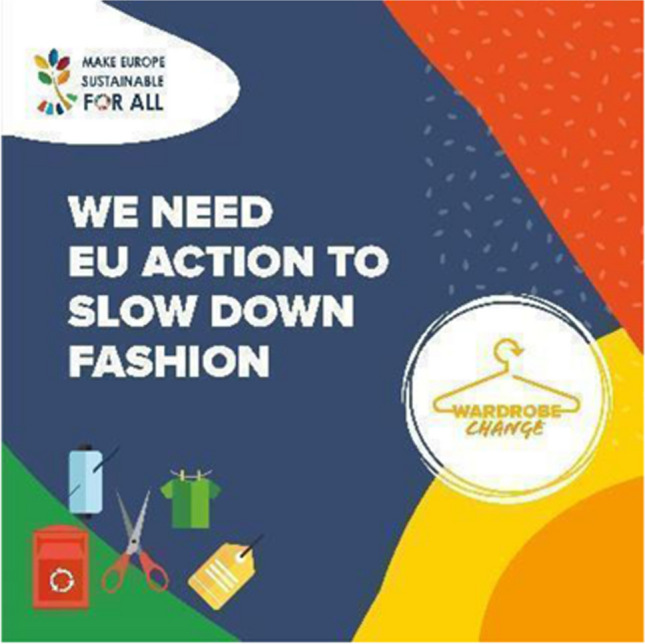
Take away message is to slow down textiles (SDG Watch Europe 2019)

We ought to correct our ways to protect our basic rights of clean air, water, earth (housing), and food (fire) and stay in synchronisation with nature (Ether aka Space) before attempting for life on Mars and Moon. Abiding by the laws of nature, biomimicry combined with the principles of green chemistry would prodigiously raise Sustainable Development Goals. Figure 21 reflects the takeaway message that is to slow down textiles.

## Future recommendations

It is imminent that prodigious efforts are required at individual level up to mass level to reinforce the sustainability in textile and fashion industries. It is imperative to abide by the United Nations Sustainable Development Goals as it is a well-designed systematic approach that does justice to entire existence on earth. Distilling from the above review, it would be obligatory to religiously follow the rituals of transparency and traceability in the textile supply chain, fair wages, and hygienic work conditions for textile factory workers; also, organic textile fibres farming would be obligatory. Phasing out of hazardous substances at all the stages of manufacturing from fibre to fabrics and installing an efficient wastewater recycling plant at textile wet processing and colouration units are mandatory. Tapping solar energy for operating textile mills, culminating inequality to BIPOC due to fast fashion landfills and occupation safety at garment manufacturing units should be essentially prioritised. Eco-friendly innovation and consumer awareness that would annihilate microfibres and over production are the need of the hour. Nevertheless, it is inescapable to connect with laws of nature and obey principles of green chemistry to reverse the climate crisis. Essentially, the Sustainable Development Goals (SDGs) extend beyond 2030 as a way of life.

## Data Availability

Authors consent to the availability of data and materials.

## References

[CR1] Akter MM, Haq UN, Islam MM, Uddin MA (2022). Textile-apparel manufacturing and material waste management in the circular economy: a conceptual model to achieve sustainable development goal (SDG) 12 for Bangladesh. Clean Environ Syst.

[CR2] Alison G (2020). A practical guide to sustainable fashion.

[CR3] Almanza AM, Corona B (2020). Using social life cycle assessment to analyse the contribution of products to the Sustainable Development Goals: a case study in the textile sector. Int J Life Cycle Assess.

[CR4] Arvind Limited (2017) Corporate social responsibility. Retrieved 08 12, 2022, from https://www.arvind.com/corporate-social-responsibility

[CR5] Banswara Syntex Limited (2016) CSR projects. Retrieved 08 18, 2022, from https://www.banswarasyntex.com/csr-projects/

[CR6] Bartlett J (2023) Fast fashion goes to die in the world's largest fog desert. The scale is breathtaking. National Geographic Partners, LLC*.* Retrieved 05.12.2023, https://www.nationalgeographic.com/environment/article/chile-fashion-pollution?rnd=1683909256714&loggedin=true

[CR7] Belzagui F, Crespi M, Álvarez A, Gutiérrez-Bouzán C, Vilaseca M (2019). Microplastics' emissions: microfibers’ detachment from textile garments. Environ Pollut.

[CR8] Biondi O, Salvatore M, Pasquale M (2002). Low molecular weight polyethylene glycol induced chromosome aberrations in Chinese hamster cells cultured in vitro. Mutagenesis.

[CR9] Burckel E (2022) Textiles fan inflation fears amid London Fashion Week. Retrieved 08 20, 2022, from https://www.barrons.com/news/textiles-fan-inflation-fears-amid-london-fashion-week-01645334407

[CR10] Cai Y-J, Choi T-M (2020). A United Nations’ Sustainable Development Goals perspective for sustainable textile and apparel supply chain management. Transp Res Part E.

[CR11] Christie RM, Mather RR, Wardman RH (2000). The chemistry of colour application.

[CR12] Cobbing M, Daaji S, Kopp M, Wohlgemuth V (2022) Poisoned gifts: from donations to the dumpsite: textiles waste disguised as second-hand clothes exported to East Africa*.* A Greenpeace Germany Report

[CR13] Cobbing M, Wohlgemuth V, Vicaire Y (2023) Greenwash danger zone. Greenpeace

[CR14] Cotton Up (2018) Why source sustainable cotton? Retrieved from Cotton Up: http://cottonupguide.org/why-source-sustainable-cotton/challenges-for-cotton/

[CR15] David Suzuki Foundation, One nature. (2022). The dirty dozen: PEG compounds and their contaminants. (David Suzuki Foundation) Retrieved 07 26, 2021, from https://davidsuzuki.org/queen-of-green/dirty-dozen-peg-compounds-contaminants/

[CR16] Debnath S, Gardetti M, Muthu S (2015). Great potential of stinging nettle for sustainable textile and fashion. Handbook of sustainable luxury textiles and fashion. Environmental Footprints and Eco-design of Products and Processes.

[CR17] Debnath S, Gardetti MA, Muthu SS (2020). Flax fibre extraction to fashion products leading towards sustainable goals. The UN Sustainable Development Goals for the textiles and fashion industry.

[CR18] Earth.Org (2021) How one community in Ghana is bearing the burden of the UK’s textile waste crisis. Retrieved 08 19, 2022, from https://earth.org/how-one-community-in-ghana-is-bearing-the-burden-of-the-uks-textile-waste-crisis/

[CR19] Ecosia (2017) Ecosia. Retrieved 08 19, 2022, from https://ecosiashop.com/

[CR20] Ellen MacArthur Foundation & Circular fibres initiatives (2017) A new textile economy: redesigning fashion’s future*.* UK: Ellen MacArthur Foundation & Circular fibres initiative

[CR21] Ethical Made Easy (2020) Ethical made easy. Retrieved 08 17, 2022, from https://ethicalmadeeasy.com/

[CR22] European Chemicals Agency. (2017) Titanium dioxide is proposed to be classified as suspected of causing cancer when inhaled. (ECHA) Retrieved 07 17, 2021, from https://echa.europa.eu/-/titanium-dioxide-proposed-to-be-classified-as-suspected-of-causing-cancer-when-inhaled

[CR23] European Food Safety Authority. (2021) Titanium dioxide: E171 is no longer considered safe when used as a food additive. (EFSA (EU)) Retrieved 07 17, 2021, from https://www.efsa.europa.eu/en/news/titanium-dioxide-e171-no-longer-considered-safe-when-used-food-additive

[CR24] Exchange T (2019). Corporate fibre and materials benchmark.

[CR25] Fashion Takes Action (2022) Fashion’s role in the Sustainable Development Goals. Retrieved 08 12, 2022, from https://fashiontakesaction.com/sdgs/

[CR26] Foreign & Commonwealth Office and Department for International Development. (2014) The Rana Plaza disaster. Retrieved 08 15, 2022, from https://www.gov.uk/government/case-studies/the-rana-plaza-disaster

[CR27] Gabriel M, Luque MLD, Gardetti M, Muthu S (2020). Sustainable Development Goal 12 and its relationship with the textile industry. The UN Sustainable Development Goals for the textile and fashion industry. Textile Science and Clothing Technology.

[CR28] Gardetti AM, Muthu SS (2020) The UN Sustainable Development Goals for the textile and fashion industry. Springer Nature Singapore Pte Ltd. 2020. ISBN : 978–981–13–8786–9. 10.1007/978-981-13-8787-6

[CR29] Gonçalves A, Silva C (2021). Looking for sustainability scoring in apparel: a review on environmental footprint, social impacts and transparency. Energies.

[CR30] Good On You (2022) Good on you. Retrieved 08 17, 2022, from https://goodonyou.eco/

[CR31] Greenpeace International (2012) Toxic threads: under wraps*.* Retrieved April 28, 2020, from https://storage.googleapis.com/planet4-international-stateless/2012/12/ToxicThreads03.pdf

[CR32] Greenpeace International (2013) Toxic threads: polluting paradise, a story of big brands and water pollution in Indonesia*.* The Netherlands: Greenpeace International. Retrieved April 29, 2020, from https://www.greenpeace.org/static/planet4-international-stateless/2013/04/62ec9171-toxic-threads-04.pdf

[CR33] Hajimirsadeghi A (n.d.) Are used clothes destroying Africa? Retrieved 08 20, 2022, from https://curiosityshots.com/are-used-clothes-destroying-africa/

[CR34] Hello Natural Living (2021) Best laundry detergents cheat sheet. Retrieved 08 21, 2022, from https://www.hellonaturalliving.com/best-laundry-detergents-cheat-sheet/#Abode_Laundry_Liquid_-_Eucalyptus

[CR35] Hempstead M (2022) Fashion and UN SDGs. Spring Wise*.* Retrieved 01, 08, 2023, from https://www.springwise.com/sustainable-source/fashion-and-the-sdgs/

[CR36] Hirsh S (2020) These companies are making vegan leather out of plants instead of plastic. Retrieved from Green Matters: https://www.greenmatters.com/p/vegan-leather-made-from-plants

[CR37] Hodal K (2018) Abuse is daily reality for female garment workers for Gap and H&M, says report*.* Guardian News & Media Limited or its affiliated companies. Retrieved 05.13.2023, from, https://www.theguardian.com/global-development/2018/jun/05/female-garment-workers-gap-hm-south-asia

[CR38] Igini M (2022) How to recognise fast fashion brands and which ones to avoid. Retrieved 08 17, 2022, from https://earth.org/fast-fashion-brands-to-avoid/

[CR39] Inditex (2022) Sustainability. Retrieved 08 20, 2022, from https://www.inditex.com/itxcomweb/en/sustainability#beyond

[CR40] Kakkar N (2018) Bandi River’s water is unfit for agricultural and domestic purposes, says the NGT monitoring committee. (Hindustan Times) Retrieved 02 01, 2022, from https://www.hindustantimes.com/jaipur/bandi-river-s-water-unfit-for-agricultural-and-domestic-purpose-says-ngt-monitoring-committee/story-s1mTKw62x8S2YqLArEhMKM.html

[CR41] Klein N (2019) No logo. Retrieved 08 19, 2022, from https://naomiklein.org/no-logo/

[CR42] Larsson JK (2018). Digital innovation for sustainability - experiences based on projects in textile value. Res J Text Appar.

[CR43] Levi Strauss & Co (2022) We're on a mission to change the clothing industry. For good. Retrieved 08 15, 2022, from https://www.levi.com/GB/en_GB/features/sustainability

[CR44] Luxiders Magazine (2022) Fashion’s future: the Sustainable Development Goals. Retrieved 08 12, 2022, from https://luxiders.com/fashions-future-the-sustainable-development-goals/

[CR45] Maiti R (2022) Fast fashion and its environmental impact. Retrieved 08 19, 2022, from https://earth.org/fast-fashions-detrimental-effect-on-the-environment/

[CR46] Malik A, Lafortune G, Carte S, Li M, Lenzen M, Kroll C (2021). International spillover effects in the EU’s textile supply chains: a global SDG assessment. J Environ Manag.

[CR47] Marrion AR (1994) The chemistry and physics of coatings. Marrion A Ed. Cambridge: The Royal Society of Chemistry

[CR48] McFall-Johnsen M (2019) The fashion industry emits more carbon than international flights and maritime shipping combined. Here are the biggest ways it impacts the planet*.* Retrieved 08 19, 2022, from https://www.businessinsider.com/fast-fashion-environmental-impact-pollution-emissions-waste-water-2019-10?r=US&IR=T#in-total-up-to-85-of-textiles-go-into-landfills-each-year-thats-enough-to-fill-the-sydney-harbor-annually-6

[CR49] Murray, Jackson (2023) Elle. 69 sustainable clothing brands that are anything but boring*.* Retrieved 08 05, 2023, from https://www.elle.com/uk/fashion/what-to-wear/g22788319/sustainable-fashion-brands-to-buy-from-now/

[CR50] Neto GC, Correia JM, Silva PC, Sanches AG, Lucato WC (2019). Cleaner production in the textile industry and its relationship to sustainable development goals. J Clean Prod.

[CR51] Nontoxic Certified (2019) Chemical callout: polyethylene glycol compounds (PEGs). (Made Safe) Retrieved 07 26, 2021, from https://www.madesafe.org/chemicalcallout-polyethylene-glycol-compounds-pegs/

[CR52] OEcotextiles (n.d.) Textile printing and the environment. (OEcotextiles) Retrieved 07 09, 2021, from https://oecotextiles.blog/2012/01/27/textile-printing-and-the-environment/

[CR53] Oldale R J (2019) The 12 Universal Laws of Nature

[CR54] Olofsson L, Mark-Herbert C (2020). Creating shared values by integrating UN Sustainable Development Goals in corporate communication—the case of apparel retail. Sustainability.

[CR55] Orange Fiber S.r.l. (2022) The first fabric from oranges. Retrieved from Orange fibre: https://orangefiber.it/tencel-limited-edition-initiative/

[CR56] Oxfam International (n.d.) 2022. Retrieved 08 15, 2022, from https://onlineshop.oxfam.org.uk/second-hand-clothes

[CR57] Patwary S (2020) Clothing and textile sustainability: current state of environmental challenges and the ways forward*.* Textile & Leather Review. http://www.textile-leather.com/tlr-3-3-2020-patwary/

[CR58] Project CECE (2016) Discover all ethical fashion in one place. Retrieved 08 17, 2022, from https://www.projectcece.com/

[CR59] Provin AP, Dutra AR, Machado MM, Cubas AL (2021). New materials for clothing: rethinking possibilities through a sustainability approach - a review. J Clean Prod.

[CR60] Radhakrishnan S (2020) Sustainable consumption and production patterns in fashion*.* Gardetti AM, Muthu SS (eds.) The UN Sustainable Development Goals for the textile and fashion industry. Springer Nature Singapore Pte Ltd. 2020. ISBN : 978–981–13–8786–9. 10.1007/978-981-13-8787-6

[CR61] Reliance Industries Limited (2022) People, planet, prosperity. Retrieved 08 20, 2022, from https://www.ril.com/Sustainability/CorporateSustainability.aspx

[CR62] Robertson L (2021) Cruelty-free fashion: 36 stylish vegan clothing brands we know you’ll love. Retrieved 08 17, 2022, from https://goodonyou.eco/vegan-fashion-brands/

[CR63] Robinson D (2021) What is greenwashing? Retrieved 08 17, 2022, from https://earth.org/what-is-greenwashing/

[CR64] Samsung Global Goals (n.d.) Learn about the Global Goals. Retrieved 08 11, 2022, from https://pwa.samsungglobalgoals.com/goals

[CR65] Sánchez LD (2020) Londoners are breathing in millions of microfibers from textiles every day. Retrieved 08 19, 2022, from https://www.oceancleanwash.org/2020/01/londoners-are-breathing-in-millions-of-microfibers-from-textiles-every-day/

[CR66] SDG Watch Austria (2020) SDGs and the textile industry – why we urgently need a #WardrobeChange. Retrieved 08 12, 2022, from https://www.sdgwatch.at/en/what-we-do/blog/2020/02/24/sdgs-and-the-textile-industry/

[CR67] SDG Watch Europe (2019) Wardrobe change. https://www.sdgwatcheurope.org/wardrobe-change/#

[CR68] Selfridges and Co (2021) Selfridges rentals. Retrieved 08 15, 2022, from https://selfridgesrental.com/

[CR69] Sharpe S, Retamal M, Brydges T (2022) Want to make your wardrobe more sustainable? Cut your new clothing purchases by 75%. (TED Conferences, LLC ) Retrieved 08 22, 2022, from https://ideas.ted.com/more-sustainable-wardrobe-cut-new-clothing-purchases-by-75-percent/

[CR70] Shore J (1990). Dye structure and application.

[CR71] Shukla N (2022) Fast fashion pollution and climate change. Retrieved 08 21, 2022, from https://earth.org/fast-fashion-pollution-and-climate-change/

[CR72] Sivasankar CA (2011) Dyeing stain on Knit City’s future. (Fibre2Fashion Pvt. Ltd) Retrieved 02 02, 2022, from https://www.fibre2fashion.com/industry-article/5695/dyeing-stain-on-knit-citys-future

[CR73] Stichting ZDHC Foundation (2022) Roadmap to zero. (Stichting ZDHC Foundation) Retrieved 09 20, 2021, from https://www.roadmaptozero.com/input

[CR74] Teemill Tech Ltd (n.d.) Rapanui. Retrieved 08 15, 2022, from https://rapanuiclothing.com/womens-custom-printed-t-shirt-dress/?stage=design-list

[CR75] Textile exchange (KPMG) (2018) Threading the needle. Weaving the Sustainable Development Goals into the textile, retail, and apparel industry

[CR76] Textile Exchange (n.d.) What are the SDGs? Retrieved 08 12, 2022, from https://textilesforsdgs.org/

[CR77] Thakker AM (2021). Sustainable processing of cotton fabrics with plant-based biomaterials Sapindus mukorossi and Acacia concinna for health-care applications. Text Inst.

[CR78] Thakker AM, Sun D (2022). Developing sustainable fabrics with plant-based formulations.

[CR79] Thakker AM, Sun D (2021). Innovative plant-based mordants and colorants for application on cotton fabric. J Nat Fibres.

[CR80] The Economist Newspaper Limited (2022) Data point: the case for circular and regenerative fashion. Retrieved 05.14.2023, from https://impact.economist.com/sustainability/circular-economies/data-point-3-the-case-for-circular-and-regenerative-fashion

[CR81] The Global Living Wage Coalition (2023) What is a living wage?.* Retrieved 05.13.2023, from* https://www.globallivingwage.org/about/what-is-a-living-wage/

[CR82] The Indian Express [P] Ltd (2021) Energy and efficiency improvement opportunities in the textile industry. Retrieved 08 21, 2022, from https://www.financialexpress.com/industry/energy-and-efficiency-improvement-opportunities-in-the-textile-industry/2355944/

[CR83] The United Nations Development Programme (UNDP) (2022) Sustainable Development Goals. Retrieved 08 19, 2022, from https://shop.undp.org/

[CR84] The United Nations Development Programme (n.d.) Global Mindpool. Retrieved 08 11, 2022, from https://globalmindpool.org/

[CR85] The United Nations Sustainable Development Goals (2022) Communication materials. Retrieved 07 15, 2021, from https://www.un.org/sustainabledevelopment/news/communications-material/

[CR86] Threadcurve.com (2021) 19 of the best online 2nd-hand clothing stores (save big). Retrieved 08 15, 2022, from https://threadcurve.com/online-second-hand-clothing-stores/#1VestiaireCollective

[CR87] UNDP, High-level Advisory Group on Sustainable Transport (2016) Mobilising sustainable transport for development. Retrieved 08 11, 2022, from https://sdgs.un.org/publications/mobilizing-sustainable-transport-development-18045

[CR88] United Nations (n.d.a) The 17 goals. Retrieved 08 02, 2023, from https://sdgs.un.org/goals

[CR89] United Nations (n.d.b) Conscious fashion and lifestyle network. Retrieved 08 02, 2023, from https://sdgs.un.org/partnerships/action-networks/conscious-fashion-and-lifestyle-network

[CR90] United Nations (n.d.c) United Nations act now. Retrieved 08 11, 2022, from https://www.un.org/en/actnow

[CR91] Veerah (2022) Apple leather. Retrieved from Veerah: https://www.veerah.com/pages/organic-apple-leather

[CR92] Vijeyarasa R, Liu M (2022). Fast fashion for 2030: using the pattern of the Sustainable Development Goals (SDGs) to cut a more gender-just fashion sector. Bus Hum Rights J.

[CR93] Vinted UAB (2022) Ready to declutter your wardrobe? Retrieved 08 15, 2022, from https://www.vinted.co.uk/

[CR94] Wolfe I (2022) These luxury brands are still harming animals for profit. Retrieved 08 17, 2022, from https://goodonyou.eco/luxury-brands-harming-animals/

[CR95] Wood J (2019). Bioinspiration in fashion—a review. Biomimetics.

[CR96] Yunus (2022) Sustainability. Retrieved 08 18, 2022, from https://www.yunustextile.com/sustainability/

